# Kinetic interacting particle Langevin Monte Carlo

**DOI:** 10.1007/s11222-026-10980-z

**Published:** 2026-09-18

**Authors:** Paul Valsecchi Oliva, O. Deniz Akyildiz

**Affiliations:** Department of Mathematics, Imperial College London, Queen’s Gate, SW7 2AZ London, UK

**Keywords:** Interacting particle Langevin algorithms, Maximum marginal likelihood estimation, Parameter inference in latent variable models

## Abstract

This paper introduces and analyses interacting underdamped Langevin algorithms, termed Kinetic Interacting Particle Langevin Monte Carlo (KIPLMC) methods, for statistical inference in latent variable models. We propose a diffusion process that evolves jointly in the space of parameters and latent variables and show that the stationary distribution of this diffusion concentrates around the maximum marginal likelihood estimate of the parameters. We then provide two explicit discretisations of this diffusion as practical algorithms to estimate parameters of statistical models. For each algorithm, we obtain nonasymptotic rates of convergence in Wasserstein-2 distance for the case where the joint log-likelihood is strongly concave with respect to latent variables and parameters. We achieve accelerated convergence rates clearly demonstrating improvement in dimension dependence. To demonstrate the utility of the introduced methodology, we provide numerical experiments that illustrate the effectiveness of the proposed diffusion for statistical inference. Our setting covers a broad number of applications, including unsupervised learning, statistical inference, and inverse problems.

## Introduction

Latent variable models (LVMs) are ubiquitous in many areas of statistical science, e.g., complex probabilistic models for text, audio, video and images (Blei et al. 2003; Smaragdis et al. 2006; Hoff et al. 2002), or inverse problems (Glyn-Davies et al. 2025). These models capture the underlying structure of the data in terms of low-dimensional variables—a structure that often exists in the real world (Whiteley et al. 2025). However, LVMs often require non-trivial procedures for their estimation as their likelihood is often intractable.

Consider a generic latent variable model as $p_\theta (x,y)$, parameterised by $\theta \in \mathbb {R}^{d_\theta }$, for *fixed*, observed data $y \in {\mathbb {R}}^{d_y}$, and latent variables $x \in {\mathbb {R}}^{d_x}$. Thus, formally, we see the statistical model as a real-valued mapping $p_\theta (x,y):\mathbb {R}^{d_x}\times \mathbb {R}^{d_\theta }\rightarrow {\mathbb {R}}$. The task we are interested in is to estimate the parameter θ that explains the fixed dataset *y*. Often, this is achieved via the maximum likelihood estimation (MLE). In our setting, due to the presence of latent variables, we aim at finding the maximum marginal likelihood estimation (MMLE), which is termed the MMLE problem (Dempster et al. 1977). More precisely, our problem takes the form

1 $$\begin{aligned} \bar{\theta }_\star \in {\mathop {\textrm{argmax}}\limits _{\theta \in \mathbb {R}^{d_\theta }}}\, \log p_\theta (y), \end{aligned}$$

where $p_\theta (y):= \int p_\theta (x,y) \textrm{d}x$ is the *marginal likelihood* (also called the model evidence in Bayesian statistics (Bernardo and Smith 2009)). It is apparent from (1) that the problem cannot be solved via optimisation techniques alone for most statistical models.

Classically, this problem is solved with the iterative expectation maximisation (EM) algorithm (Dempster et al. 1977), which increases the marginal likelihood at every iteration and, under regularity conditions, converges to a stationary point of $\theta \mapsto \log p_\theta (y)$ (Wu 1983). At a given parameter estimate $\theta _n$, the EM algorithm implements a map $\theta _n \!\mapsto \! {\textrm{argmax}}_{\theta \!\in \! \mathbb {R}^{d_\theta }} \mathbb {E}_{p_{\theta _n} (x|y)}[\log p_{\theta } (x, y)]$, which is guaranteed not to decrease the marginal likelihood at any iteration. This procedure requires an “E-step”, namely the computation (or estimate) of the expectation w.r.t. the posterior distribution of the latent variables $p_{\theta _n}(x|y)$, and an “M-step”, which is the maximisation of the expected log-likelihood w.r.t. θ. These steps are intractable in general and can be approximated with a variety of methods, which have been extensively explored. For example, Monte Carlo EM (Wei and Tanner 1990) and stochastic EM (Celeux 1985) have been widely studied (Celeux and Diebolt 1992; Chan and Ledolter 1995; Sherman et al. 1999; Booth and Hobert 1999; Cappé et al. 1999; Diebolt and Ip 1996).

In the most general case, the EM algorithm is implemented using Markov chain Monte Carlo (MCMC) techniques for the E-step (Atchadé et al. 2017), and numerical optimisation techniques for the M-step (Meng and Rubin 1993; Liu and Rubin 1994; Lange 1995). With the popularity of unadjusted Langevin algorithm (ULA) in Bayesian statistics and machine learning (Durmus and Moulines 2017, 2019; Dalalyan 2017), novel variants of EM algorithms use unadjusted chains. Most notably, De Bortoli et al. (2021) studied an algorithm termed Stochastic Optimisation via Unadjusted Langevin (SOUL), which performs unadjusted Langevin steps for the E-step and stochastic gradient ascent for the M-step, building on the ideas of Atchadé et al. (2017). In the MCMC based EM setting, the bias incurred *by using finitely many* MCMC steps complicates the theoretical analysis and requires stringent conditions for step-sizes.

An alternative approach was developed in Kuntz et al. (2023) where the authors proposed an interacting particle system, consisting of *N* particles to replace sequential MCMC steps. This framework, termed particle gradient descent (PGD), is based on the idea of joint optimisation and sampling to avoid a double-loop structure as in MCMC-based approaches, which results in an efficient algorithmic framework as well as one that is amenable to theoretical analysis Caprio et al. (2025). Inspired by the approach in Kuntz et al. (2023), a closely related interacting particle system was proposed in Akyildiz et al. (2025), where a scaled noise is injected in the θ-dimension. The authors termed this method interacting particle Langevin algorithm (IPLA) and proved error bounds for the algorithm. This seemingly small modification is significant, making the algorithm an instance of a Langevin diffusion (an observation we build on in this paper), streamlining the theoretical analysis and opening the door to a wide variety of extensions with theoretical guarantees. The IPLA methodology has been already utilised in other contexts, see, Johnston et al. (2025) for superlinear extensions and Cordero-Encinar et al. (2025) for a set of proximal methods based on IPLA, and Akyildiz et al. (2024) for relations between IPLA and multiscale methods.

**Contributions.** In this paper, we develop optimisation methods for MMLE based on underdamped Langevin samplers. Our contributions can be summarised as follows:

**(C1)** We propose the kinetic interacting particle Langevin diffusion (KIPLD), a diffusion process to optimise the marginal likelihood in latent variable models. We prove that the stationary measure of this diffusion concentrates around the MMLE (Propositions 1 and 2) and the KIPLD converges to this measure exponentially fast (Proposition 3).

**(C2)** We then develop our first kinetic interacting particle Langevin Monte Carlo (KIPLMC) method, termed KIPLMC1—an exponential integrator discretisation of KIPLD. We prove discretisation error bounds for our method showing convergence rates in both time and step-size (Theorem 1), closing the problem of nonasymptotic analysis of this method in the strongly convex case. In particular, we show that KIPLMC1 attains accelerated rates of convergence compared to its overdamped counterparts IPLA (Akyildiz et al. 2025) and PGD (Caprio et al. 2025). While these overdamped diffusion-based MMLE methods achieve an ε error in Wasserstein-2 distance in $\widetilde{\mathcal {O}}(d_x \varepsilon ^{-2})$ steps, we prove that KIPLMC1 attains ε error in $\widetilde{\mathcal {O}}(\sqrt{d_x} \varepsilon ^{-1})$ steps.

**(C3)** We then introduce a splitting-based explicit discretisation scheme within this setting, which leads to a novel MMLE algorithm, termed KIPLMC2. We study the nonasymptotic behaviour of this method (Theorem 2), showing that the algorithm obtains an error ε in Wasserstein-2 distance in $\widetilde{\mathcal {O}}(d_\theta \sqrt{d_x}\,\varepsilon ^{-3})$ steps.

**(C4)** Finally, we provide solid empirical evidence of the performance of our methods in a variety of settings, including synthetic and realistic models. In the convex setting, we validate the $\mathcal {O}(N^{-1/2})$ concentration onto the maximiser $\bar{\theta }_\star$ and demonstrate the increased stability of the momentum-based methods. In the non-convex setting, we show the acceleration of the proposed methods, as well as the importance of the choice of numerical integrator, validating our methodological contribution.

**Relations to existing work.** The KIPLD is similar to the continuous time system on which the momentum particle gradient descent (MPGD) algorithm by Lim et al. (2024) is based (see Eq. (21) in Lim et al. (2024) where we take $\eta _\theta = \eta _x = 1$ and $\gamma _x = \gamma _\theta = \gamma$). In line with the work done by Akyildiz et al. (2025), we inject the θ-dynamics with an appropriately scaled Brownian motion, i.e., $\sqrt{2\gamma /N} {\textrm{d}}{{\textbf{B}}}^0_t$. The advantage of studying and implementing this version is due to the fact that the new system can be shown to be an example of a standard underdamped Langevin diffusion, which enables us to show non-asymptotic bounds in a streamlined manner. Furthermore, our discretisations differ from the MPGD routine: the KIPLMC1 algorithm does not use a gradient correction step, whilst KIPLMC2 is based on a different integrator. In Sect. 5, we show that these algorithms obtain similar stability, while having theoretical guarantees.

The paper is structured as follows. In Sect. 2, we present the background work on underdamped Langevin diffusions for sampling and interacting particle systems for the MMLE problem. Following this introduction, in Sect. 3, we present a new diffusion targeting the solution of the MMLE problem and present the associated algorithms KIPLMC1 and KIPLMC2. Section 4 presents the nonasymptotic analysis of KIPLMC methods, providing a clear evidence of improved theoretical bounds due to the acceleration. Finally, in Sect. 5, we present a variety of experiments to support our theoretical findings.

### Notation

Let $\mathcal {P}(\mathbb {R}^d)$, for d≥1, denote the set of probability measures on $(\mathbb {R}^d,\mathcal {B}(\mathbb {R}^d))$, where $\mathcal {B}(\mathbb {R}^d)$ is the Borel σ-algebra. We write $\langle \cdot ,\cdot \rangle$ and $\Vert \cdot \Vert$ for the Euclidean inner product and norm on $\mathbb {R}^d$. We also use $[N]=\{1,\dots ,N\}$ and ${\mathbb {N}}$ for the positive integers. For p≥1, define the Wasserstein-*p* distance by

$$ W_p(\pi ,\nu ) = \left( \inf _{\Gamma \in \textbf{T}(\pi , \nu )} \int _{\mathbb {R}^d\times \mathbb {R}^d} \Vert x-y\Vert ^p\,\textrm{d}\Gamma (x,y)\right) ^{1/p}, $$

where $\textbf{T}(\pi ,\nu )$ denotes the set of couplings of π and ν, i.e., probability measures Γ on $\mathbb {R}^d\times \mathbb {R}^d$ with marginals π and ν. We also define $\widetilde{\mathcal {O}}$ as the big $\mathcal {O}$ notation, up to logarithmic orders.

## Technical background

Before presenting our proposed diffusion and algorithms, we introduce some concepts that will be useful to us in this paper. We first introduce the kinetic Langevin diffusion and its associated algorithms, which are the building blocks of our proposed methods. Then, we present the IPLA algorithm, which is the closest method to our proposed algorithms and serves as a starting point for our developments.

### Kinetic Langevin Monte Carlo

In recent years, the Langevin diffusion and its associated algorithms have been widely used in statistics and machine learning to sample from complex distributions. The overdamped Langevin diffusion is given by the stochastic differential equation (SDE)

2 $$\begin{aligned} \textrm{d}{{\textbf{Z}}}_t = -\nabla U({{\textbf{Z}}}_t) \textrm{d}t + \sqrt{2}\textrm{d}{{\textbf{B}}}_t, \end{aligned}$$

where $U:{\mathbb {R}}^d \rightarrow {\mathbb {R}}$ is a potential function and $({{\textbf{B}}}_t)_{t\ge 0}$ is a Brownian motion. Under certain regularity conditions, this system is known to be invariant w.r.t. the measure $\pi ({\textrm{d}}z) \propto \exp (-U(z)){\textrm{d}}z$ (Pavliotis 2014). Under the strongly convex and Lipschitz-gradient setting, a standard Euler-Maruyama discretisation of this diffusion, called ULA, is known to attain at most $\mathcal {O}(\varepsilon )$ error in Wasserstein-2 distance to the stationary measure in $\widetilde{\mathcal {O}}(d \varepsilon ^{-2})$ steps (Dalalyan 2017; Durmus and Moulines 2019).

An alternative to the *overdamped* Langevin diffusions given in (2), is another class of diffusions called *underdamped* Langevin diffusions (Pavliotis 2014). This class of diffusions is akin to second-order differential equations and defined over position and momentum variables. In particular, for a *d*-dimensional target measure $\pi ({\textrm{d}}z) \propto \exp (-U(z)){\textrm{d}}z$, the underdamped (kinetic) Langevin diffusion is given by the SDE

3 $$\begin{aligned} \begin{aligned} \textrm{d}{{\textbf{Z}}}_t&= {{\textbf{V}}}_t \textrm{d}t \\ \textrm{d}{{\textbf{V}}}_t&= - \gamma {{\textbf{V}}}_t \textrm{d}t - \nabla _z U({{\textbf{Z}}}_t)\textrm{d}t + \sqrt{2\gamma }\textrm{d}{{\textbf{B}}}_t, \end{aligned} \end{aligned}$$

where γ>0 is called the friction coefficient and $({{\textbf{B}}}_t)_{t \ge 0}$ is a Brownian motion. Under certain regularity conditions (Pavliotis 2014), this system is known to be invariant w.r.t. an extended stationary measure of the form

4 $$\begin{aligned} \bar{\pi }({\textrm{d}}z, {\textrm{d}}v) \propto \exp \left( -U(z) - \frac{1}{2}\Vert v\Vert ^2\right) {\textrm{d}}z {\textrm{d}}v. \end{aligned}$$

This means that we can recover the samples from our target measure $\pi ({\textrm{d}}z) \propto \exp (-U(z)){\textrm{d}}z$ by sampling from (4) and then marginalising out the velocity variable.

The structure in (3) allows for smoother sample paths and faster convergence to the stationary measure compared to the overdamped case (Cheng et al. 2018). This has motivated the development of algorithms based on discretisations of the underdamped Langevin diffusion, termed kinetic Langevin Monte Carlo (KLMC) (Dalalyan and Riou-Durand 2020). These methods have been shown to achieve improved convergence rates compared to their overdamped counterparts, making them a competitive alternative for sampling from complex distributions. In particular, under the strongly convex and Lipschitz-gradient setting, KLMC is known to attain at most $\mathcal {O}(\varepsilon )$ error in Wasserstein-2 distance to the stationary measure in $\widetilde{\mathcal {O}}(\sqrt{d} \varepsilon ^{-1})$ steps (Dalalyan and Riou-Durand 2020). In this paper, we build on the framework of KLMC methods to develop algorithms for the MMLE problem.

### MMLE via interacting particle Langevin algorithm

Consider the MMLE problem given in (1), that is, to find the parameter θ that maximises the marginal likelihood $p_\theta (y)$. As summarised in the introduction, this problem is often solved with the EM algorithm, typically implemented with a double-loop structure consisting of MCMC techniques for the E-step and numerical optimisation techniques for the M-step (Atchadé et al. 2017; De Bortoli et al. 2021).

In contrast to this approach, Kuntz et al. (2023) propose an interacting particle system, the PGD algorithm, to approximate the gradient of the θ-dynamics by running independent particles to integrate out latent variables. Inspired by this, Akyildiz et al. (2025) attempt to solve the MMLE problem with a similar system, termed the IPLA, which is a modification of PGD where θ-dynamics contain a carefully scaled noise. This approach produces a system that is akin to the ULA and makes the theoretical analysis streamlined using the analysis produced for ULA (Dalalyan 2017; Durmus and Moulines 2019). The proposed algorithm is a discretisation of a system of interacting Langevin SDEs which evolves in ${\mathbb {R}}^{d_\theta } \times {\mathbb {R}}^{N d_x}$ where *N* distinct particles are retained for latent variables. More precisely, IPLA recursions are based on the SDE:

5 $$\begin{aligned} \textrm{d}{\boldsymbol{\theta }}_t&= -\frac{1}{N} \sum _{i=1}^N \nabla _\theta U({\boldsymbol{\theta }}_t, {{\textbf{X}}}_t^{i}) \textrm{d}t + \sqrt{\frac{2}{N}}\textrm{d}{{\textbf{B}}}_t^0, \end{aligned}$$

6 $$\begin{aligned} \textrm{d}{{\textbf{X}}}_t^{i}&= -\nabla _x U({\boldsymbol{\theta }}_t, {{\textbf{X}}}^i_t)\textrm{d}t + \sqrt{2}\textrm{d}{{\textbf{B}}}_t^i, \end{aligned}$$

for i∈[N], where $({{\textbf{B}}}_t^0)_{t\ge 0}$ is a Brownian motion evolving on ${\mathbb {R}}^{d_\theta }$ and $({{\textbf{B}}}_t^i)_{t\ge 0}$ for i∈[N] are Brownian motions evolving on $\mathbb {R}^{d_x}$. In this case, Akyildiz et al. (2025) observe that the θ-marginal of the stationary measure of this system takes the form $\pi _\Theta ({\textrm{d}}\theta ) \propto \exp (N \log p_\theta (y)) {\textrm{d}}\theta$ which concentrates on the MMLE $\bar{\theta }_\star$ as *N* grows, where *N* plays the role of inverse temperature (Hwang 1980). The authors then identify the convergence rate to the joint stationary measure, as well as an error bound for the discretisation error, from which an error can be determined between the θ-iterate of the algorithm and the MMLE.

## Kinetic interacting particle Langevin Monte Carlo

To develop our methodology, we start with the following diffusion process:

**Figure:**
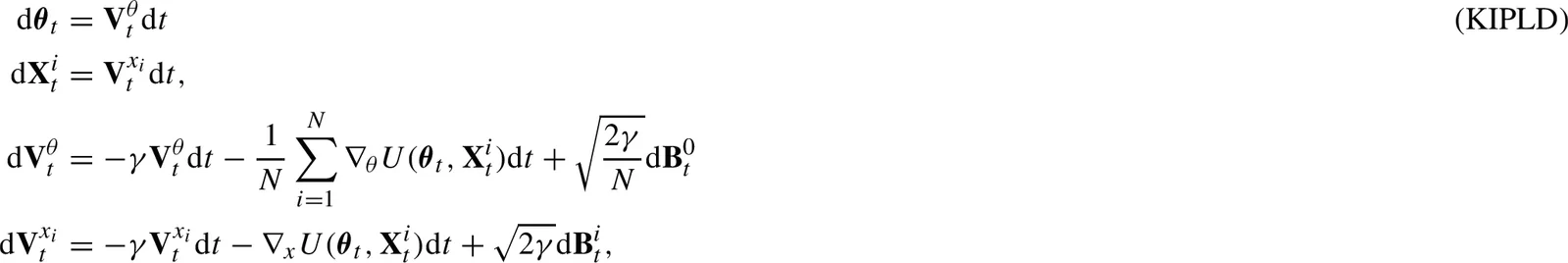

for i∈[N], where $\{({{\textbf{B}}}_t^i)_{t\ge 0}\}_{i\in [N]}$ is a family of ${\mathbb {R}}^{d_x}$-valued Brownian motions and $({{\textbf{B}}}_t^0)_{t\ge 0}$ is an $\mathbb {R}^{d_\theta }$-valued Brownian motion. This diffusion has the property that its θ-marginal at stationarity concentrates around the global maximisers of $\log p_\theta (y)$ (see Sect. 4).

Next, we introduce two numerical integrators for the KIPLD: firstly, we consider an Exponential Integrator, as discussed in Dalalyan and Riou-Durand (2020); secondly, a splitting scheme is applied, as described in Monmarché (2021).

### Exponential integrator (KIPLMC1)

In order to derive the exponential integrator discretisation of KIPLD, following Dalalyan and Riou-Durand (2020), we begin by defining the functions

$$\begin{aligned} \psi _0^t&= e^{-\gamma t},\\ \psi _1^t&= \int _0^t e^{-\gamma s} {\textrm{d}}s = \frac{1}{\gamma }(1-e^{-\gamma t}),\\ \psi _2^t&= \int _0^t \psi _1^s {\textrm{d}}s = \frac{1}{\gamma ^2}(e^{-\gamma t}-1) + \frac{t}{\gamma }. \end{aligned}$$

The exponential integrator is obtained by freezing the nonlinear drift terms $\nabla _\theta U(\theta _t,X_t^i)$ and $\nabla _x U(\theta _t,X_t^i)$ at the beginning of each time step and solving exactly the resulting linear stochastic differential equation.

More precisely, let $t_n=n\eta$. Over the interval $[t_n,t_{n+1}]$ we approximate the dynamics (KIPLD) by freezing the gradients at $(\theta _n,X_n^i)$,

$$ F_n^\theta = \frac{1}{N}\sum _{i=1}^N \nabla _\theta U(\theta _n,X_n^i), \quad F_n^{x_i} = \nabla _x U(\theta _n,X_n^i), $$

which yields the linear system

$$\begin{aligned} \textrm{d}{\boldsymbol{\theta }}_t&= {{\textbf{V}}}_t^\theta \textrm{d}t, \\ \textrm{d}{{\textbf{X}}}_t^i&= {{\textbf{V}}}_t^{x_i} \textrm{d}t,\\ \textrm{d}{{\textbf{V}}}_t^\theta&= -\gamma {{\textbf{V}}}_t^\theta \textrm{d}t - F_n^\theta \textrm{d}t + \sqrt{\frac{2\gamma }{N}}\textrm{d}{{\textbf{B}}}_t^0,\\ \textrm{d}{{\textbf{V}}}_t^{x_i}&= -\gamma {{\textbf{V}}}_t^{x_i} \textrm{d}t - F_n^{x_i} \textrm{d}t + \sqrt{2\gamma }\textrm{d}{{\textbf{B}}}_t^i, \end{aligned}$$

for i∈[N], with initial condition

$$ ({\boldsymbol{\theta }}_{t_n},{{\textbf{X}}}_{t_n}^i,{{\textbf{V}}}_{t_n}^\theta ,{{\textbf{V}}}_{t_n}^{x_i}) = (\theta _n,X_n^i,V_n^\theta ,V_n^{x_i}). $$

Since the system is linear with constant coefficients, it can be solved explicitly. Using an integrating factor for the velocity equation gives, for $s\in [0,\eta ]$,

$$\begin{aligned} V_{t_n+s}^\theta&= \psi _0^s V_n^\theta - \psi _1^s F_n^\theta + \sqrt{\frac{2\gamma }{N}} \int _0^s \psi _0^{\,s-\tau }\,\textrm{d}{{\textbf{B}}}_{t_n+\tau }^0,\\ V_{t_n+s}^{x_i}&= \psi _0^s V_n^{x_i} - \psi _1^s F_n^{x_i} + \sqrt{2\gamma } \int _0^s \psi _0^{\,s-\tau }\,\textrm{d}{{\textbf{B}}}_{t_n+\tau }^i. \end{aligned}$$

Integrating once more yields the updates for the positions

$$\begin{aligned} \theta _{t_n+s}&= \theta _n + \psi _1^s V_n^\theta - \psi _2^s F_n^\theta + \sqrt{\frac{2\gamma }{N}} \int _0^s \psi _1^{\,s-\tau }\,\textrm{d}{{\textbf{B}}}_{t_n+\tau }^0,\\ X_{t_n+s}^i&= X_n^i + \psi _1^s V_n^{x_i} - \psi _2^s F_n^{x_i} + \sqrt{2\gamma } \int _0^s \psi _1^{\,s-\tau }\,\textrm{d}{{\textbf{B}}}_{t_n+\tau }^i. \end{aligned}$$

Evaluating these expressions at s=η gives the one-step update of the discretised process. The stochastic integrals appearing above are jointly Gaussian random variables. For each $i\in \{0,\ldots ,N\}$ we define

$$\begin{aligned} \varepsilon _n^i = \int _0^\eta \psi _0^{\,\eta -s}\,\textrm{d}B_{t_n+s}^i, \quad \varepsilon _n^{i,\prime } = \int _0^\eta \psi _1^{\,\eta -s}\,\textrm{d}B_{t_n+s}^i. \end{aligned}$$

**Algorithm 1:**
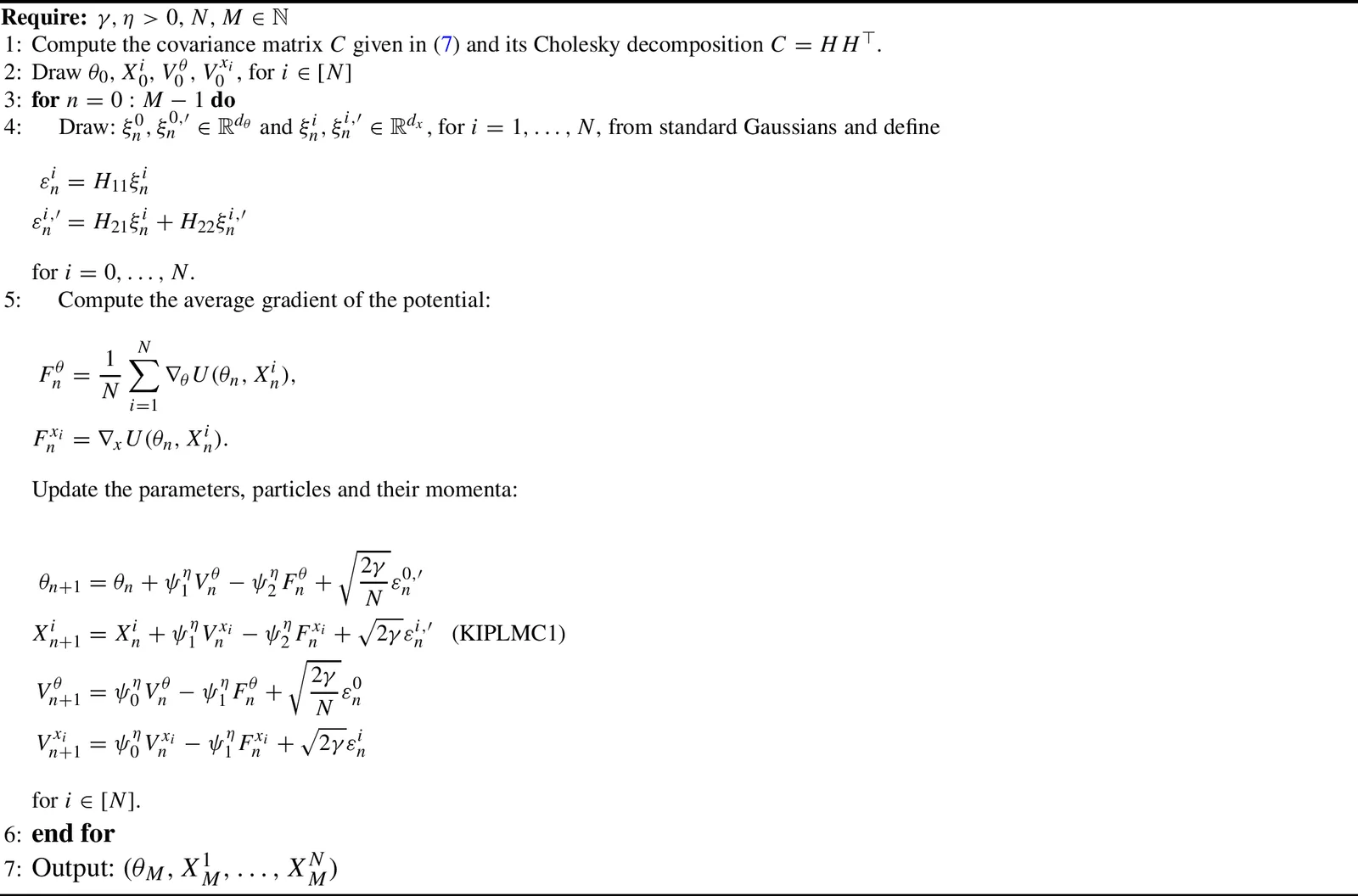
KIPLMC1

Now note that $(\varepsilon _n^0, \varepsilon _n^{0,\prime }) \in \mathbb {R}^{d_\theta } \times \mathbb {R}^{d_\theta }$ and $(\varepsilon _n^i, \varepsilon _n^{i,\prime }) \in \mathbb {R}^{d_x} \times \mathbb {R}^{d_x}$ for i=1,…,N. Consider each coordinate of these pairs:

$$\begin{aligned} \varepsilon _{n,j}^i&= \int _0^\eta \psi _0^{\,\eta -s}\,\textrm{d}B_{t_n+s,j}^i,\\ \varepsilon _{n,j}^{i,\prime }&= \int _0^\eta \psi _1^{\,\eta -s}\,\textrm{d}B_{t_n+s,j}^i. \end{aligned}$$

For every (*i*, *j*), using Itô isometry (Oksendal 2013), we can obtain $C = \textrm{Cov}(\varepsilon _{n,j}^i, \varepsilon _{n,j}^{i,\prime })$ as

7 $$\begin{aligned} C = \int _0^\eta \begin{pmatrix} (\psi _0^{t})^2 & \psi _0^t\psi _1^t\\ \psi _0^t\psi _1^t & (\psi _1^{t})^2 \end{pmatrix} \textrm{d}t. \end{aligned}$$

We note that $C\in \mathbb {R}^{2\times 2}$ and denote its Cholesky decomposition by $C=H H^\top$. Hence the Gaussian pair $(\varepsilon _{n,j}^i, \varepsilon _{n,j}^{i,\prime })$ can be sampled by setting

$$\begin{aligned} \begin{pmatrix}\varepsilon _{n,j}^i\\ \varepsilon _{n,j}^{i,\prime }\end{pmatrix}&= H \begin{pmatrix}\xi _{n,j}^i\\ \xi _{n,j}^{i,\prime }\end{pmatrix}, \end{aligned}$$

where $\xi _{n,j}^i$ and $\xi _{n,j}^{i,\prime }$ are independent standard Gaussian random variables.

Substituting these expressions for s=η yields the KIPLMC1 scheme. The full algorithm is given in Algorithm 1 and a more explicit derivation in Appendix B.

### A splitting scheme (KIPLMC2)

We next introduce KIPLMC2, an adaptation of the underdamped Langevin sampler introduced by Horowitz (1991) and extensively studied by Monmarché (2021). This splitting scheme is termed OBABO and it is a second order scheme, only requiring the first order derivatives of *U*. The idea is to split the numerical scheme into simpler subflows that can be solved explicitly. More precisely, in our KIPLD case, we decompose the flow into a Ornstein-Uhlenbeck step (O), a velocity drift step (B) and a particle update step (A).

In the OBABO case, over a full time-step η, the (O) step is applied over a η/2 half-step, the (B) step is also applied to a η/2 half-step, (A) is applied as a full time-step η, followed by a reversed application of (B) and (O) for half-steps. We denote these intermediate time-steps by $n+\frac{1}{4}$, $n+\frac{1}{2}$ and $n+\frac{3}{4}$, though we note that these time-steps do not correspond to simulated time. The order in which these steps are taken is critical (Monmarché 2021) and in our case we will limit ourselves to considering the OBABO ordering.

More precisely, the Ornstein-Uhlenbeck half-step (O) flow is given as

$$\begin{aligned} \textrm{d} {{\textbf{V}}}_t^\theta&= -\gamma {{\textbf{V}}}_t^\theta \textrm{d}t + \sqrt{\frac{2\gamma }{N}} \textrm{d}{{\textbf{B}}}_t^0,\\ \textrm{d} {{\textbf{V}}}_t^{x_i}&= - \gamma {{\textbf{V}}}_t^{x_i} \textrm{d} t + \sqrt{2\gamma }\textrm{d}{{\textbf{B}}}_t^i, \end{aligned}$$

over a half-step of duration η/2, for i∈[N], where the parameters ${\boldsymbol{\theta }}_t$ and ${{\textbf{X}}}_t^i$ are frozen and we initialise at $({{\textbf{V}}}_{n\eta }^\theta , {{\textbf{V}}}_{n\eta }^{x_i})= (V_n^\theta , V_n^{x_i})$. Using the classic analytic solution to the Ornstein-Uhlenbeck equation and the Itô Isometry we obtain,

$$\begin{aligned} \begin{aligned} V_{n+\frac{1}{4}}^\theta&= \delta V_n^\theta + \frac{1}{\sqrt{N}} \sqrt{1-\delta ^2} \xi _n^0,\\ V_{n+\frac{1}{4}}^{x_i}&= \delta V_n^{x_i} + \sqrt{1-\delta ^2} \xi _n^i, \quad \quad \quad \quad \quad \text {(O)} \end{aligned} \end{aligned}$$

with $\delta =e^{-\eta \gamma /2}$ and i.i.d. standard Gaussians $\xi _n^0$ and $\xi _n^i$, in $\mathbb {R}^{d_\theta }$ and $\mathbb {R}^{d_x}$, respectively.

The momentum drift update (B) updates $$V^\theta $$ and $$V^{x_i}$$ by exactly integrating the flow with frozen gradients $$N^{-1} \sum _{i=1}^N$$$$\nabla _\theta U(\theta , X^i)$$ and $$\nabla _{x_i}U(\theta , X^i)$$, i.e. we consider the flow to be given as

$$\begin{aligned} \textrm{d} {{\textbf{V}}}_t^\theta&= - F_n^\theta \textrm{d}t,\\ \textrm{d} {{\textbf{V}}}_t^{x_i}&= - F_n^{x_i} \textrm{d}t, \end{aligned}$$

**Algorithm 2:**
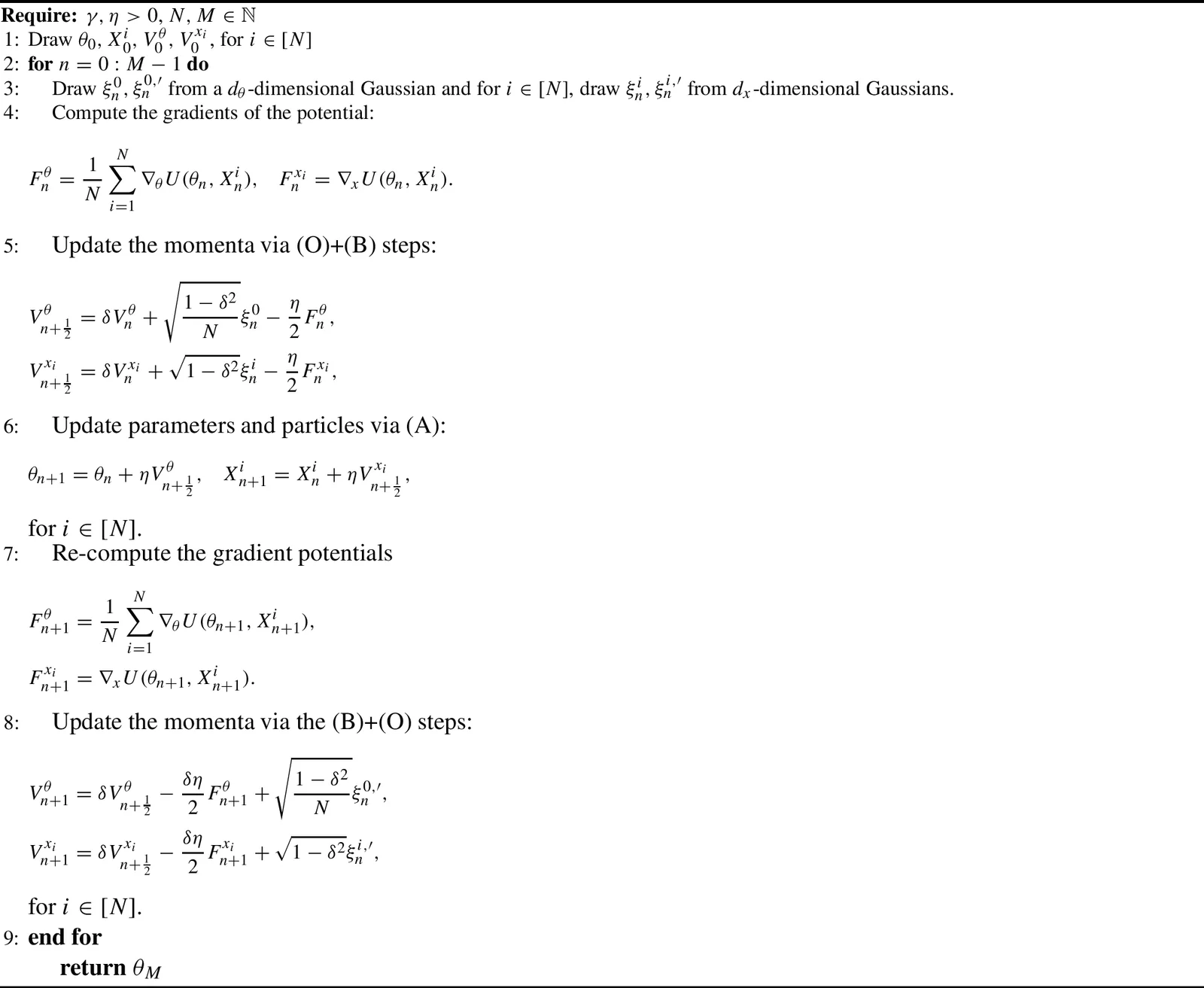
KIPLMC2 Algorithm

over a half-step of duration η/2, where we recall the definitions of $F_n^\theta$ and $F_n^{x_i}$ from Sect. 3.1 and initialise at $(V_{n+\frac{1}{4}}^\theta ,V_{n+\frac{1}{4}}^{x_i})$, the output of the (O) half-step. This yields the exact update:

$$\begin{aligned} \begin{aligned} V_{n+\frac{1}{2}}^\theta&= V_{n+\frac{1}{4}}^\theta - \frac{\eta }{2} F_n^\theta ,\\ V_{n+\frac{1}{2}}^{x_i}&= V_{n+\frac{1}{4}}^{x_i} - \frac{\eta }{2} F_n^{x_i}.\quad \quad \quad \quad \quad \text {(B)} \end{aligned} \end{aligned}$$

Finally, the (A) update considers the partial flow,

$$\begin{aligned} \textrm{d} {\boldsymbol{\theta }}_t&= {{\textbf{V}}}_t^\theta \textrm{d}t,\\ \textrm{d} {{\textbf{X}}}_t^i&= {{\textbf{V}}}_t^{x_i}\textrm{d}t, \end{aligned}$$

over $t\in [t_n, t_{n+1}]$, where we consider $({{\textbf{V}}}_t^\theta ,{{\textbf{V}}}_t^{x_i})$ is frozen at $(V_{n+1/2}^\theta , V_{n+1/2}^{x_i})$ and we initialise at $({\boldsymbol{\theta }}_{n\eta }, {{\textbf{X}}}_{n\eta }^i)=(\theta _n, X_n^i)$. Hence, the (A) update is given as,

$$\begin{aligned} \begin{aligned} \theta _{n+1}&= \theta _n + \eta V_{n+\frac{1}{2}}^\theta ,\\ X_{n+1}^i&= X_n^i + \eta V_{n+\frac{1}{2}}^{x_i}.\quad \quad \quad \quad \quad \text {(A)} \end{aligned} \end{aligned}$$

The algorithm is then completed by repeating steps (B) and (O),

$$\begin{aligned} \begin{aligned} V_{n+1}^\theta&= \delta \left( V_{n+\frac{1}{2}}^\theta -\frac{\eta }{2} F_{n+1}^\theta \right) + \sqrt{\frac{1-\delta ^2}{N}} \xi _n^{0,\prime },\\ V_{n+1}^{x_i}&= \delta \left( V_{n+\frac{1}{2}}^{x_i} - \frac{\eta }{2} F_{n+1}^{x_i}\right) + \sqrt{1-\delta ^2} \xi _n^{i,\prime }, \quad \quad \text {(B+O)} \end{aligned} \end{aligned}$$

where the (B) and (O) subflows are each applied over a half-step of duration η/2, and where $\xi _n^{0,\prime }$ and $\xi _n^{i,\prime }$ are standard Gaussians in $\mathbb {R}^{d_\theta }$ and $\mathbb {R}^{d_x}$, respectively.

The full updates are given in Algorithm 2 and a full derivation of the KIPLMC2 algorithm is given in Appendix B.

## Nonasymptotic analysis

In this section, we provide the convergence results for both the analytic and numerical schemes in the nonasymptotic regime. In Sect. 4.1, we lay out our assumptions about our target measure. In Sect. 4.2, we introduce the proof strategy for the reader’s convenience, which may be useful for extending and proving similar results. In Sect. 4.3, we provide the full nonasymptotic bounds—in particular, in Sect. 4.3.1, we identify the stationary measure for KIPLD and show an exponential convergence rate to it, as well as, its concentration onto the MMLE solution $\bar{\theta }_\star$ as *N* grows. Following this, in Sect. 4.3.2, the error bounds for the two algorithms are provided, allowing us to identify the convergence rate of KIPLMC1 and KIPLMC2 to the MMLE solution $\bar{\theta }_\star$.

### Assumptions

Our assumptions are generic for the analysis of Langevin diffusions, i.e., we assume strong convexity and *L*-Lipschitz gradients for the potential *U*. These assumptions are akin to the assumptions made in Durmus and Moulines (2017), Dalalyan (2017), Dalalyan and Karagulyan (2019) for proving the convergence of ULA. It is, however, possible to relax them, as can be seen in Zhang et al. (2023), Akyildiz and Sabanis (2024).

We start with the following assumption on the potential *U*.

#### H 1

Suppose $U\in C^2(\mathbb {R}^{d_\theta +d_x})$. There exists a constant μ>0 such that, for all $z,z'\in \mathbb {R}^{d_\theta +d_x}$,

$$\begin{aligned} \langle z-z', \nabla U(z) - \nabla U(z')\rangle \ge \mu \Vert z-z'\Vert ^2. \end{aligned}$$

Since $U\in C^2$, this is equivalent to $\nabla ^2 U(z)\succeq \mu I$ for all $z\in \mathbb {R}^{d_\theta +d_x}$, i.e., *U* is μ-strongly convex.

This assumption is equivalent to an assumption of joint strong convexity in θ and *x* for *U*, which ensures exponentially fast convergence of the KIPLD to the global minimiser and places a quadratic lower growth bound on *U*.

#### H 2

There exists a constant L>0 such that, for all $z,z'\in \mathbb {R}^{d_\theta +d_x}$,

$$\begin{aligned} \Vert \nabla U(z) - \nabla U(z')\Vert \le L \Vert z-z'\Vert . \end{aligned}$$

Together with Assumption **H**1, this implies $\mu \le L$. This assumption is a common assumption in the analysis of stochastic differential equations, as it ensures stability of the diffusion KIPLD, leading to strong solutions, and stable numerical discretisations (Kloeden et al. 1993). Observe that this assumption places an upper quadratic growth bound on *U* or, from the perspective of the probabilistic model, $\log p_\theta (x,y)$ is of quadratic growth in both θ and *x*. **H**1 and **H**2 together ensure that we can apply the Leibniz differentiation rule under integration for the marginal of the probability model $p_\theta (x, y)= e^{-U(\theta ,x)}$ ((Billingsley 1995), Theorem 16.8).

We remark nonetheless that, in the works cited above, the assumptions are on the strong log-concavity of the target measure. In our setting, this corresponds to imposing a structure on the joint statistical model $p_\theta (x, y)$ in θ and *x*, as our potential is given as $U(\theta ,x) = -\log p_\theta (x,y)$. This requires some care on the user side as a statistical model with strongly log-concave posterior $p_\theta (x | y)$ (with fixed θ) may not necessarily satisfy strong log-concavity of $p_\theta (x, y)$ in (x,θ) jointly. We verify in the experimental section that for some generic models such as Bayesian logistic regression, strict convexity can be shown (which can be improved to strong convexity with regularisation). As noted above, however, these strong assumptions on the statistical model should not be difficult to relax using the standard non-log-concave sampling results—but this direction is out of scope of the present work which considers the strongly convex case.

### The proof strategy

Let $(\theta _n)_{n\ge 0}$ be the sequence of iterates generated by a numerical scheme, for example, KIPLMC1 or KIPLMC2. Let $\pi _\Theta$ denote the θ-marginal of the stationary measure of the diffusion KIPLD, and $\delta _{\bar{\theta }_\star }$ denote the Dirac measure at $\bar{\theta }_\star$. Finally, we denote the law of $\theta _n$ by $\mathcal {L}(\theta _n)$.

In order to proceed, we first note that the optimisation error is given as ${\mathbb {E}}[\Vert \theta _n - \bar{\theta }_\star \Vert ^2]^{1/2} = W_2(\mathcal {L}(\theta _n), \delta _{\bar{\theta }_\star })$. This is due to the fact that the set of couplings between a measure ν and another measure $\delta _y$ contains a single element $\delta _y \otimes \nu$ ((Santambrogio 2015), Section 1.4), thus the infimum in Wasserstein-2 distance is attained by the coupling $\delta _{\bar{\theta }_\star } \otimes \mathcal {L}(\theta _n)$. This implies that ${\mathbb {E}}[\Vert \theta _n - \bar{\theta }_\star \Vert ^2]^{1/2} = W_2(\mathcal {L}(\theta _n), \delta _{\bar{\theta }_\star })$ and consequently using triangle inequality

8 $$\begin{aligned} {\mathbb {E}}[\Vert \theta _n - \bar{\theta }_\star \Vert ^2]^{1/2} \le \underbrace{W_2(\mathcal {L}(\theta _n), \pi _\Theta )}_{\text {convergence}} + \underbrace{W_2(\pi _\Theta , \delta _{\bar{\theta }_\star })}_{\text {concentration}}, \end{aligned}$$

as the Wasserstein distance is a metric. The first term is the convergence of the numerical scheme to the stationary measure $\pi _\Theta$ which will be proved for each scheme separately. The second term in the right-hand side of (8) is the concentration of the stationary measure $\pi _\Theta$ onto the MMLE solution $\bar{\theta }_\star$, which is given by Proposition 2 below.

### Convergence bounds

As outlined above in (8), we will first show the concentration of the stationary measure $\pi _\Theta$ onto the MMLE solution $\bar{\theta }_\star$, then show the convergence of the KIPLD to the stationary measure $\pi _\Theta$. Finally, we will provide the error bounds for the numerical schemes.

#### Concentration of the stationary measure

This is a non-standard underdamped Langevin diffusion, thus, we need to first identify the stationary measure of the system. Recall that we are only interested in θ-marginal of this stationary measure and we denote it by $\pi _\Theta$. We first have the following proposition.

##### Proposition 1

Let $\pi _\Theta$ be the θ-marginal of the stationary measure of the KIPLD. Then, we can write its density as

9 $$\begin{aligned} \pi _\Theta (\theta ) \propto \exp ( - N \kappa (\theta )). \end{aligned}$$

where $$\kappa (\theta ) = - \log p_\theta (y)$$.

##### Proof

See Appendix C.1. □

This result shows that the KIPLD targets the right object: as *N* grows, $\pi _\Theta$ will concentrate on the minimisers of $\kappa (\theta )$ by a classical result (Hwang 1980). This shows that the number of particles *N* acts as an inverse temperature parameter in the underdamped diffusion. Since the minimiser of $\kappa (\theta )$ is the maximiser of $\log p_\theta (y)$, we can see that the stationary measure of the KIPLD is concentrating on the MMLE solution $\bar{\theta }_\star$. In particular, under the assumption **H**1, we have the following nonasymptotic concentration result.

##### Proposition 2

Under **H**1, the function $\theta \mapsto p_\theta (y)$ is μ-strongly log-concave. Furthermore, we have

$$\begin{aligned} W_2(\pi _\Theta , \delta _{\bar{\theta }_\star })\le \sqrt{\frac{d_\theta }{\mu N}}, \end{aligned}$$

where $\bar{\theta }_\star = {\mathop {\textrm{argmax}}\limits _{\theta \in \mathbb {R}^{d_\theta }}}\, \log p_\theta (y)$, which is unique.

##### Proof

See Appendix C.2. □

We note here that this bound is tight, as it is achieved when $\pi _\Theta$ is Gaussian.

#### Convergence of the KIPLD to the stationary measure

We next demonstrate that the KIPLD converges to its stationary measure $\pi _\Theta$ exponentially fast in the strongly log-concave case.

##### Proposition 3

Let $({\boldsymbol{\theta }}_t)_{t\ge 0}$ be the θ-marginal of a solution to the KIPLD, initialised so that

$$\begin{aligned} {{\textbf{Z}}}_0 :=({\boldsymbol{\theta }}_0,N^{-1/2}{{\textbf{X}}}_0^1,\ldots ,N^{-1/2}{{\textbf{X}}}_0^N)^\intercal \sim \nu , \end{aligned}$$

where $\nu \in \mathcal {P}(\mathbb {R}^{d_z})$ has finite second moment and $d_z=d_\theta +Nd_x$. Independently of ${{\textbf{Z}}}_0$, initialise the mutually independent velocities as

$$\begin{aligned} {{\textbf{V}}}_0^\theta&\sim \mathcal {N}(0,N^{-1}\mathbb {I}_{d_\theta }),\\ {{\textbf{V}}}_0^{x_i}&\sim \mathcal {N}(0,\mathbb {I}_{d_x}),\qquad i\in [N]. \end{aligned}$$

Then, under **H**1 and **H**2 and for $\gamma \ge \sqrt{\mu + L}$, for every t≥0 we have

$$\begin{aligned} W_2(\mathcal {L}({\boldsymbol{\theta }}_t), \pi _\Theta ) \le \sqrt{2} \exp \left( -\frac{\mu }{\gamma }t\right) \mathbb {E}[\Vert {{\textbf{Z}}}_0 - \bar{Z}_\star \Vert ^2]^{1/2}, \end{aligned}$$

where $\bar{Z}_\star \sim {\widetilde{\pi }}_Z$, the position marginal of the invariant measure ${\widetilde{\pi }}$ in Lemma A.1, is drawn independently of ${{\textbf{Z}}}_0$.

##### Proof

See Appendix C.3. □

#### Nonasymptotic analysis of KIPLMC1

In this section we present our first main result, the convergence rate of KIPLMC1.

##### Theorem 1

Let $(\theta _n)_{n\in {\mathbb {N}}}$ be the iterates of KIPLMC1 and suppose that the process is initialised as $Z_0\sim \nu$, where $\nu \in \mathcal {P}(\mathbb {R}^{d_z})$ has bounded second moments, $V^\theta _0\sim \mathcal {N}(0, N^{-1}\mathbb {I}_{d_\theta })$ and $V^{x_i}_0 \sim \mathcal {N}(0, \mathbb {I}_{d_x})$, for i=1,⋯,N. Under Assumptions **H**1 and **H**2 and with $\gamma \ge \sqrt{\mu +L}$ and $\eta \le \mu /(4\gamma L)$, we have

$$\begin{aligned} {\mathbb {E}}[\Vert \theta _n - \bar{\theta }_\star \Vert ^2]^{1/2}&\le C_1 \left( 1-\frac{0.75\mu \eta }{\gamma }\right) ^n + \eta C_2 + \sqrt{\frac{C_3}{N}}, \end{aligned}$$

where

$$\begin{aligned} C_1&= \sqrt{2} {\mathbb {E}}[\Vert Z_0 - \bar{Z}_\star \Vert ^2]^{1/2}, \\ C_2&= \sqrt{2}\frac{L}{\mu } \sqrt{\frac{d_\theta + N d_x}{N}}, \\ C_3&= \frac{d_\theta }{\mu }, \end{aligned}$$

where $Z_0=(\theta _0, N^{-1/2} X^1_0,\dots , N^{-1/2} X^N_0)^\intercal$, the initialisation step of the KIPLMC1 where $\bar{Z}_\star \sim {\widetilde{\pi }}_Z$ and ${\widetilde{\pi }}_Z$ is the position marginal of the invariant measure ${\widetilde{\pi }}$ described in Lemma A.1.

##### Proof

See Appendix C.4. □

To provide the algorithmic complexity of KIPLMC1, we can inspect the bound provided in Theorem 1. To obtain a complexity result, we first set $N = \mathcal {O}(d_\theta \varepsilon ^{-2})$ which ensures the last term in the bound in Theorem 1 is $\mathcal {O}(\varepsilon )$. Next, set $\eta = \mathcal {O}(\varepsilon d_x^{-1/2})$ which ensures that the second term is $\mathcal {O}(\varepsilon )$. It is then easy to see that $n = \widetilde{\mathcal {O}}(\varepsilon ^{-1} d_x^{1/2})$ steps are enough to obtain ${\mathbb {E}}[\Vert \theta _n - \bar{\theta }_\star \Vert ^2]^{1/2} \le \varepsilon$.

##### Remark 1

This dependency needs to be compared with IPLA and PGD. For the former, we note that our dependence to dimension for *N* is identical to IPLA and the dependence of the number of steps is significantly improved, i.e., we require $n = \widetilde{\mathcal {O}}({d_x}^{1/2} \varepsilon ^{-1})$ whereas IPLA requires $n = \widetilde{\mathcal {O}}({d_x} \varepsilon ^{-2})$ steps for the same accuracy. Regarding the latter method, PGD, our dependence on the number of particles is different, as PGD requires $N = \mathcal {O}(d_x \varepsilon ^{-2})$ (but this difference is a more general difference stemming from the different analysis techniques, as IPLA also has the same dependence as us to the number of particles *N*, see, e.g., Corollary 8 and the subsequent discussion in Caprio et al. (2025)). However, in terms of the number of steps necessary to attain ε-error, we have the same improvement in dimension dependence in $d_x$ and ε as we have compared to IPLA.

#### Nonasymptotic analysis of KIPLMC2

We present our second main result, showing the convergence rate of KIPLMC2.

##### Theorem 2

Let $(\theta _n)_{n\in \mathbb {N}}$ be the iterates of KIPLMC2, set $d_z=d_\theta +Nd_x$, and define the rescaled initial position

$$\begin{aligned} Z_0=(\theta _0,N^{-1/2}X_0^1,\ldots ,N^{-1/2}X_0^N)^\intercal . \end{aligned}$$

Suppose that $Z_0\sim \nu$, where $\nu \in \mathcal {P}(\mathbb {R}^{d_z})$ has finite second moment, and, independently of $Z_0$, initialise the mutually independent velocities as

$$\begin{aligned} V_0^\theta&\sim \mathcal {N}(0,N^{-1}\mathbb {I}_{d_\theta }),\\ V_0^{x_i}&\sim \mathcal {N}(0,\mathbb {I}_{d_x}),\qquad i\in [N]. \end{aligned}$$

Under Assumptions **H**1 and **H**2, if $\gamma \ge 2\sqrt{L}$ and $0<\eta \le \mu /(33\gamma ^3)$, then, for every $n\in \mathbb {N}$, letting ${\widetilde{\pi }}_Z$ denote the position marginal of the invariant measure ${\widetilde{\pi }}$ in Lemma A.1, we have

$$\begin{aligned} {\mathbb {E}}[\Vert \theta _n-\bar{\theta }_\star \Vert ^2]^{1/2}&\lesssim \sqrt{N}\,W_2(\nu ,{\widetilde{\pi }}_Z) \exp \left( -\frac{\mu \eta n}{6\gamma }\right) \\&\quad +\eta N\sqrt{d_\theta +Nd_x} \exp \left( -\frac{\mu \eta n}{6\gamma }\right) \\&\quad +\eta \sqrt{N(d_\theta +Nd_x)} +\sqrt{\frac{d_\theta }{N}}. \end{aligned}$$

The implicit constant depends only on μ, *L*, and γ; in particular, it is independent of *n*, *N*, η, $d_\theta$, and $d_x$.

##### Proof

See Appendix C.5. □

To obtain complexity, similarly to the analysis provided for KIPLMC1, we first set $N\asymp d_\theta \varepsilon ^{-2}$ to obtain an $\mathcal {O}(\varepsilon )$ bound for the last term in Theorem 2. With this choice,

$$\begin{aligned} \sqrt{N(d_\theta +Nd_x)} \asymp \frac{d_\theta }{\varepsilon } \sqrt{1+\frac{d_x}{\varepsilon ^2}}. \end{aligned}$$

We therefore choose

$$\begin{aligned} \eta&\asymp \min \left\{ \frac{\mu }{33\gamma ^3}, \frac{\varepsilon }{\sqrt{N(d_\theta +Nd_x)}}\right\} \\&\asymp \min \left\{ \frac{\mu }{33\gamma ^3}, \frac{\varepsilon ^2}{d_\theta \sqrt{1+d_x/\varepsilon ^2}}\right\} , \end{aligned}$$

which makes the nondecaying discretisation term of order ε. In the small-ε regime, this gives $\eta =\mathcal {O}(\varepsilon ^3/(d_\theta \sqrt{d_x}))$. Finally, writing $D_0:=W_2(\nu ,{\widetilde{\pi }}_Z)$ and taking

$$\begin{aligned} n\gtrsim \frac{1}{\eta } \log \left( 1+\frac{\sqrt{N}\,D_0 +\eta N\sqrt{d_\theta +Nd_x}}{\varepsilon }\right) \end{aligned}$$

controls both exponentially decaying terms. Hence, assuming that the initial Wasserstein distance grows at most polynomially in *N* and the dimensions, $n=\widetilde{\mathcal {O}}(d_\theta \sqrt{d_x}\,\varepsilon ^{-3})$ steps are sufficient to obtain an accuracy of order ε, where the notation also suppresses factors depending only on μ, *L*, and γ.

**Table 1:** Comparison of orders required to achieve an error of order ε in $W_2$, for step count, particle number *N* and step size η

	Step count (*n*)	*N*	η
KIPLMC1	$\widetilde{\mathcal {O}}(\sqrt{d_x}/\varepsilon )$	$\mathcal {O}(d_\theta /\varepsilon ^2)$	$\mathcal {O}(\varepsilon /\sqrt{d_x})$
KIPLMC2	$\widetilde{\mathcal {O}}(d_\theta \sqrt{d_x}/\varepsilon ^3)$	$\mathcal {O}(d_\theta /\varepsilon ^2)$	$\mathcal {O}(\varepsilon ^3/(d_\theta \sqrt{d_x}))$
IPLA	$\widetilde{\mathcal {O}}(d_x/ \varepsilon ^{2})$	$\mathcal {O}(d_\theta /\varepsilon ^2)$	$\mathcal {O}(\varepsilon ^2/d_x)$
PGD	$\widetilde{\mathcal {O}}(d_x/\varepsilon ^2)$	$\mathcal {O}(d_x/\varepsilon ^2)$	$\mathcal {O}(\varepsilon ^2 /d_x)$

A few comments are in order for this complexity analysis for KIPLMC2.

##### Remark 2

In general, to avoid poor dependence on *N*, the error bounds of numerical discretisations must depend on *L* and μ with the matching powers. This ensures that the factors cancel out appropriately, preventing any undesirable effects. This is achieved by the analysis of the exponential integrator in Dalalyan and Riou-Durand (2020) with a linear dependence on the condition number L/μ, thus our KIPLMC1 bound does not have a bad dependence to *N*. This enables us to obtain improved algorithmic complexity as discussed in Remark 1. However, for KIPLMC2, the OBABO scheme does not have this dependence on the condition number (see, e.g., Monmarché (2021, Table 2)) under just **H**1 and **H**2. This results in a bound in Theorem 2 that grows as *N* grows, however, we note that it is the authors’ belief that this dependence may be an artifact of the proof method employed, rather than reflecting a deteriorating accuracy with growing *N* for KIPLMC2. This is observed in the numerical experiments we present where increasing *N* does not lead to a growing error.

See Table 1 for a summary of our discussion.

## Experiments

**Fig. 1:**
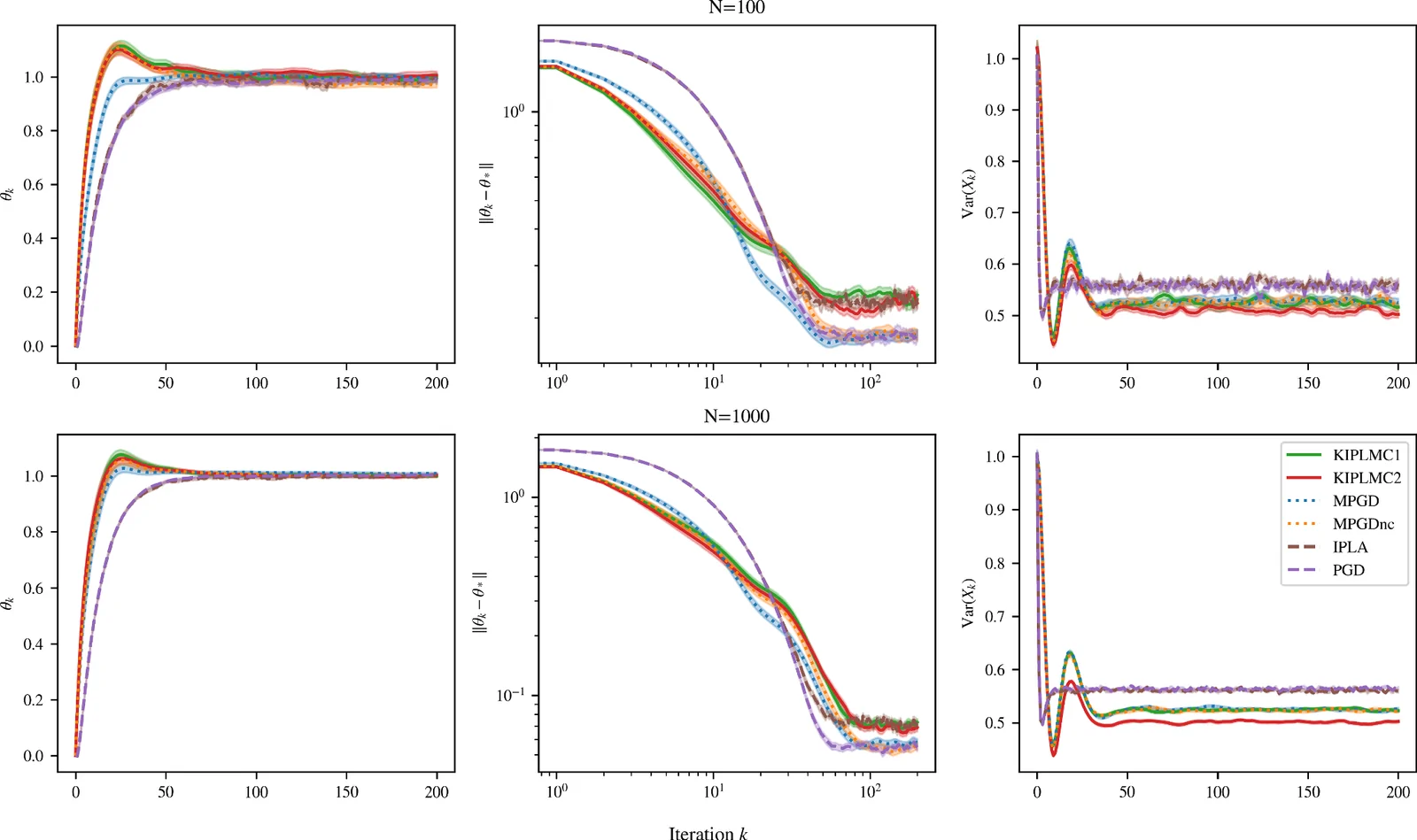
In the submitted manuscript the figures are spread out to match their reference, whilst in the new Experiments section they appear much earlier. We believe it helps the presentation to have the figures closer to their first reference and have them more spread out. We would appreciate if it would be possible to match the original submission presentation as closely as possible. Parameter estimate comparison. We compare the performance of the PGD, IPLA, MPGD, KIPLMC1, and KIPLMC2 algorithms on the synthetic dataset with true $\bar{\theta }_\star = \mathbb {1}_{d_x}$. We observe the desired convergence of behaviours for larger values of *N* in the parameter space, mean-square error and the posterior variance of $X_k^i$. In this example $d_x=d_\theta =3$, $d_y=100$ and γ=2.2. Here we choose $\theta _0, X_0^i, V^\theta _0, V^{x_i}_0\sim \mathcal {N}(0, 0.1)$ and run 100 Monte Carlo simulations

In the following section a comparison will be made between the empirical results of KIPLMC1, KIPLMC2 algorithms, as well as, those of PGD from Kuntz et al. (2023), IPLA from Akyildiz et al. (2025) and MPGD from Lim et al. (2024). We note that, in Lim et al. (2024), the MPGD is not solely the discretisation of the KIPLD-like SDE using the exponential integrator, but includes a *gradient correction* term, making theoretical analysis and a direct comparison with our methods non-trivial (for a comparison of computational cost see Sect. D.2). To this end, we will also compare these methods to MPGDnc, the MPGD algorithm implemented without gradient correction, so that the number of gradient computations is the same as KIPLMC1.

In the following sections, we will consider three examples: (i) Bayesian logistic regression on a synthetic dataset, (ii) Bayesian logistic regression on the Wisconsin Cancer dataset, and (iii) a Bayesian Neural Network (BNN) example as a more challenging, non-convex example on the MNIST dataset.

**Fig. 2:**
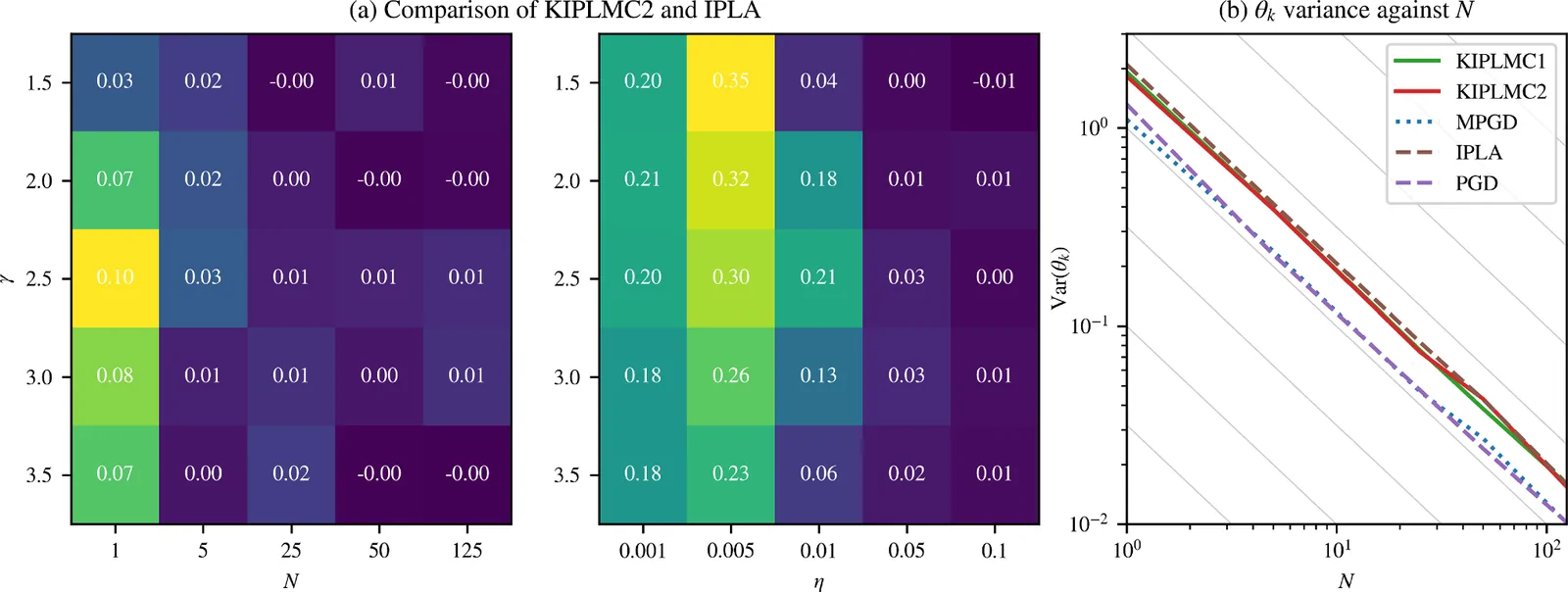
Comparison over hyper-parameters. **a** shows the Area Between the Curves (ABC) values between IPLA and KIPLMC2 for a variety of hyper-parameter combinations. The larger the positive value, the greater the acceleration of KIPLMC2 with respect to IPLA (discussed in C.1). **b** makes a comparison over 100 Monte Carlo simulations of the algorithms’ sample variances, using the last 100 steps of each simulation. The grid-lines corresponding to 1/*N* are provided to highlight the rate of convergence. Where not specified otherwise, simulations are run with M=2000, η=0.005, γ=2.2 and N=100

### Bayesian logistic regression on synthetic data

We follow the experimental setting in Kuntz et al. (2023) and Akyildiz et al. (2025) and start with comparisons between algorithms on a synthetic dataset for which we know the true solution. More precisely, consider the Bayesian logistic regression model

$$\begin{aligned} p_\theta (x)&= \mathcal {N}(x; \theta , \sigma ^2 I_{d_x})\\ p(y|x)&= \prod _{j=1}^{d_y} s(v_j^\intercal x)^{y_j} (1-s(v_j^\intercal x))^{1-y_j}. \end{aligned}$$

Here $s(u)=e^u/(1+e^u)$ is the logistic function and $v_j$, $j\in [d_y]$ are the set of $d_x$-dimensional covariates with corresponding responses $y_j\in \{0,1\}$. σ is given and fixed throughout. We generate a synthetic set of covariates, $\{v_j\}_{j=1}^{d_y}\subset \mathbb {R}^{d_x}$, from which are simulated a synthetic set of observations $y_j|\bar{\theta }_\star , x, v_j$, for fixed $\bar{\theta }_\star$, via a Bernoulli random variable with probability $s(v_j^\intercal x)$. The algorithm is tested on the recovery of this value of $\bar{\theta }_\star$.

The marginal likelihood is given as,

$$\begin{aligned}&p_\theta (y)\\&= \int \left( \prod _{j=1}^{d_y} s(v_j^\intercal x)^{y_j} (1-s(v_j^\intercal x))^{1-y_j} \right) p_\theta (x)\textrm{d}x. \end{aligned}$$

From this it easy to see that the gradients of *U* are given as,

10 $$\begin{aligned} \begin{aligned} \nabla _\theta U(\theta , x)&= -\frac{x-\theta }{\sigma ^2},\\ \nabla _x U(\theta ,x)&= \frac{x-\theta }{\sigma ^2} - \sum _{j=1}^{d_y} (y_j - s(v_j^\intercal x))v_j. \end{aligned} \end{aligned}$$

#### Remark 3

*(On* **H**1*and* **H**2*)* We will discuss this problem and our assumptions. Writing z=(θ,x) and $z^{\prime }=\left(\theta^{\prime },x^{\prime }\right)$, using $$\Vert (a-b,-a+b)\Vert \le 2\Vert (a,b)\Vert $$ and the fact that the logistic function is 1/4-Lipschitz, we obtain

$$\begin{aligned} \begin{aligned} \Vert \nabla U(z)-\nabla U(z')\Vert&\le \left( \frac{2}{\sigma ^2}+\frac{1}{4}\sum _{j=1}^{d_y}\Vert v_j\Vert ^2\right) \Vert z-z'\Vert . \end{aligned} \end{aligned}$$

Hence, **H**2 is satisfied (Akyildiz et al. 2025).

For **H**1, note that $d_\theta =d_x$ in this model. Set $q_j(x):=s(v_j^\intercal x)\bigl (1-s(v_j^\intercal x)\bigr )$ and $Q(x):=\sum _{j=1}^{d_y}q_j(x)v_jv_j^\intercal$. Then

$$\begin{aligned} \begin{aligned} \nabla ^2U(z)&=\frac{1}{\sigma ^2} \begin{pmatrix} I_{d_x}& -I_{d_x}\\ -I_{d_x}& I_{d_x} \end{pmatrix} +\begin{pmatrix} 0& 0\\ 0& Q(x) \end{pmatrix}. \end{aligned} \end{aligned}$$

Indeed, for $u,w\in \mathbb {R}^{d_x}$, $(u, w)^\intercal \nabla ^2U(z)(u,w)$ vanishes if and only if u=w and $v_j^\intercal w=0$ for every *j*. Thus, *U* is strictly convex provided that $\operatorname {span}\{v_1,\ldots ,v_{d_y}\}=\mathbb {R}^{d_x}$. Consequently, **H**1 is not satisfied without further regularisation; nevertheless, we believe that this remains a useful illustrative example.

**Fig. 3:**
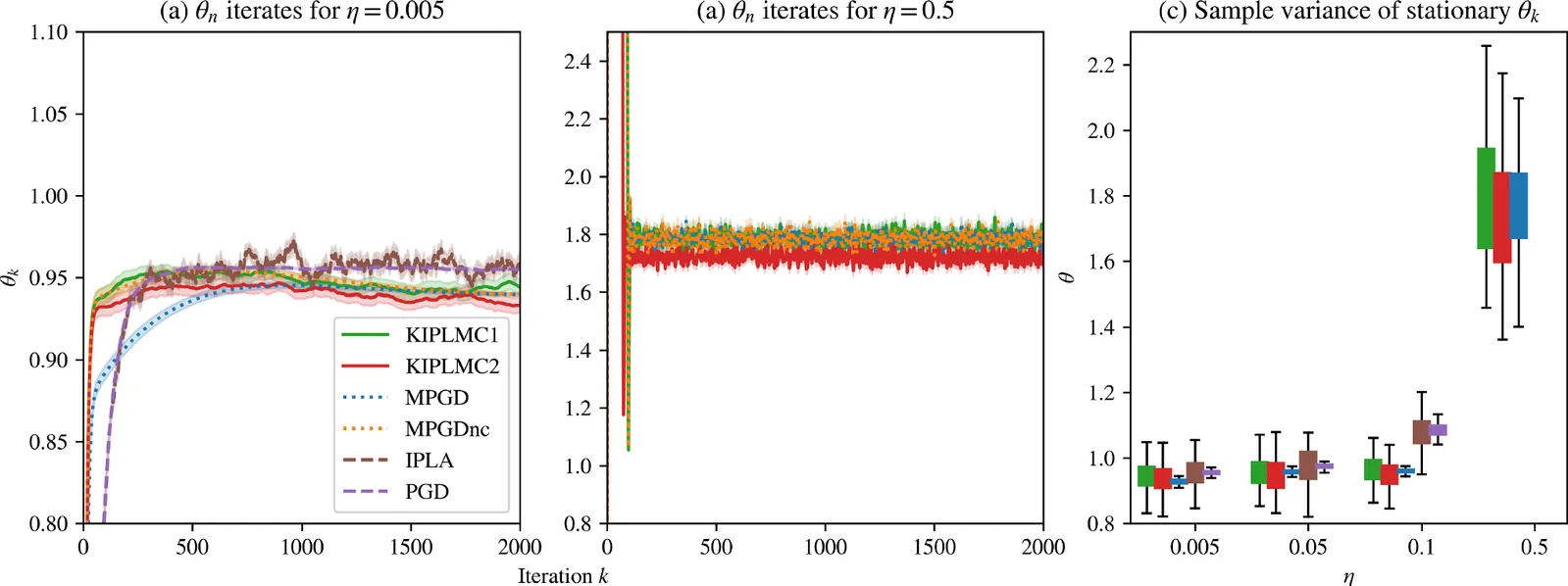
Wisconsin Dataset. The performance of PGD, IPLA, MPGD, KIPLMC1 and KIPLMC2 algorithms are compared on a logistic regression experiment for the Wisconsin Cancer Dataset. In **a**, we show the behaviour of the $\theta _n$ iterates for a small step-size and **b** shows the behaviour $\theta _n$ iterates for a step-size where IPLA and PGD explode. In **c** we compare the distributions of the algorithms over different step-sizes. We set N=100 and γ=3.2

In Fig. 1 we can see the difference in the behaviours between the algorithms. Most notably, the KIPLMC1 and KIPLMC2 algorithms exhibit comparable levels of acceleration as the MPGD algorithm in the θ-dimension, whilst the IPLA and PGD algorithms trail further behind. Interestingly, we observe (more notably for large *N*) that the noise in the θ-dynamics in KIPLMC1 and KIPLMC2, as compared to MPGD, seems to dampen some of the momentum effects when γ, the friction coefficient is sub-optimal. As *N* grows the θ iterates concentrate onto the MMLE $\bar{\theta }_\star$ for all algorithms. This behaviour can be seen in more detail in Fig. 2b, in which we can verify the variance changing with rate $\mathcal {O}(1/N)$. Further, we make a comparison of the performance of the IPLA and KIPLMC2 in Fig. 2a with the Area Between the Curves (ABC) metric (see D.3 for more detail). This metric is a signed, weighted difference between the performances of the two algorithms: the higher the value, the better KIPLMC2 performs compared to IPLA; 0 is when they perform the same. We observe that the acceleration of KIPLMC2 can be best observed in specific γ regimes, which ensure that the momentum is close to critical dampening.

Note that the momentum effects of the KIPLMC1 and KIPLMC2 algorithms has been dampened through a specific choice of γ. In training it was observed that the convergence rate is very sensitive to the choice of γ, where choices too small, exhibit large momentum effects, and too large, lead to slow convergence (see also Lim et al. 2024 for more discussion on the role of the momentum parameter for learning with KLMC). The choice of γ here is far from optimal, but allows us to observe the strength of the proposed algorithms.

### Wisconsin cancer data

We follow again an experimental procedure that is similar to the one outlined in Kuntz et al. (2023) and Akyildiz et al. (2025) and make comparisons between the algorithms on a more realistic dataset: the Wisconsin Cancer Data. Again, we use the logistic regression LVM model, outlined above. This task is a binary classification, to determine from 9 features gathered from tumours and 683 data points labelled as either benign or malignant. The latent variables correspond to the features extracted from the data. The task in this case is to seek to model the behaviour as accurately as possible through the logistic regression LVM.

For this setup we define our probability model as,

$$\begin{aligned} p_\theta (x) = \mathcal {N}(x;\theta {1}_{d_x}, 5 I_{d_x}) \end{aligned}$$

and the likelihood as,

$$\begin{aligned} p(y|x)=\prod _{j=1}^{d_y} s(v_j^\intercal x)^{y_j}(1-s(v_j^\intercal x))^{1-y_j}. \end{aligned}$$

Note that the parameter θ is a scalar in this case, hence, this turns out to be a simplified version of the setup described above and so the discussion in Remark 3 is still valid. As opposed to the previous case with synthetic data, here we consider a real dataset, so no ground-truth $\bar{\theta }^\star$ is available. Hence, there is no comparison to a $\bar{\theta }_\star$, but we can see that different algorithms attain similar estimates of this maximiser.

**Fig. 4:**
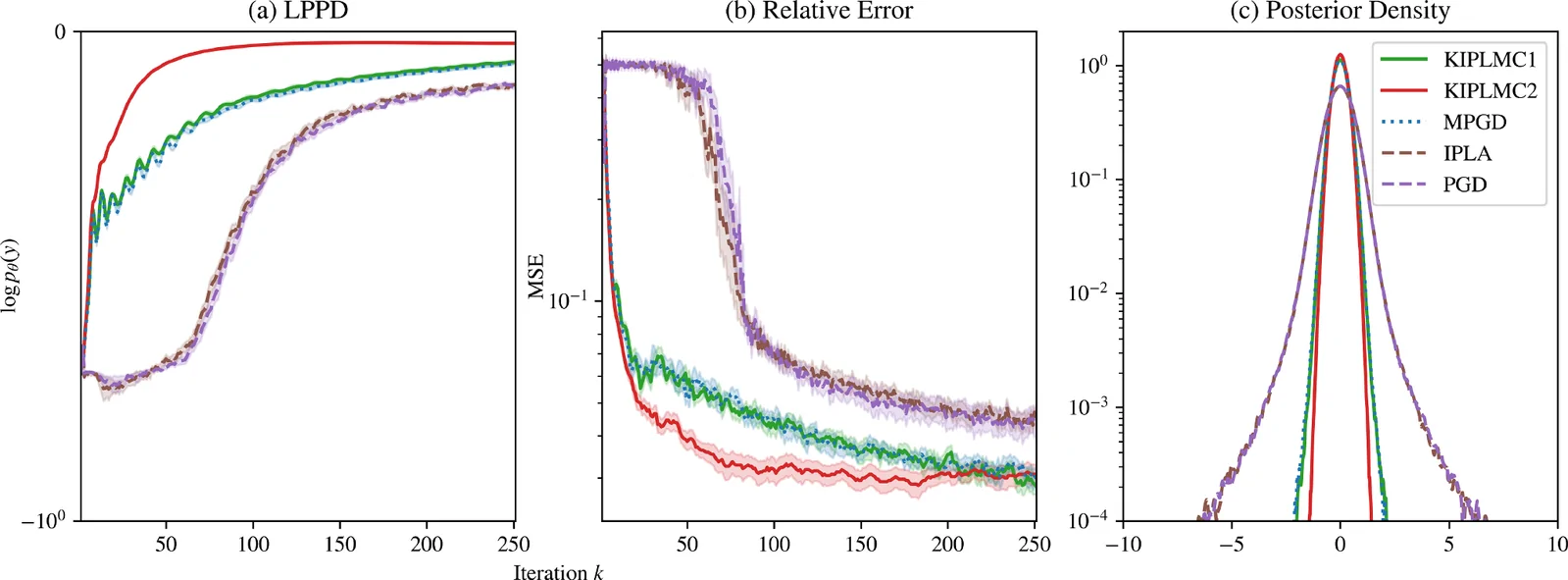
MNIST Dataset. The performance of PGD, IPLA, MPGD, KIPLMC1 and KIPLMC2 algorithms are compared on a classification experiment for the MNIST Dataset. **a** Shows the Log Pointwise Predictive Density, the average log probability assigned to the correct response. **b** Shows the percentage error and **c** the posterior density of the weights *w*. For this experiment N=100, γ=2 and η=0.01

Most notably in this experiment, we can observe in Fig. 3 the importance of the added stability that the momentum-based algorithms exhibit, with the use of more stable numerical integrators than Euler-Maruyama. In particular, these algorithms displays great stability w.r.t. the choice of the step-size, as well as, the acceleration one would expect. For small step-sizes, the PGD algorithm performs with low variance in all cases where it converges, until it explodes where step-sizes become too large. It is interesting to note however that in the large step-size regime, the momentum-based algorithms exhibit much greater momentum than in the smaller step-sizes. However, again, we note that the KIPLMC1 and KIPLMC2 algorithms exhibit more variance in the θ estimation than the PGD and MPGD algorithms, though it is damped when compared to the IPLA algorithm, due to the higher regularity of the solutions to KIPLD. This is typically an advantage when working with convex problems, but this “sticky” behaviour might prove detrimental in the non-convex case (Gao et al. 2022; Akyildiz et al. 2025). The injection of noise into the parameter estimation may help the method to escape local minima (Akyildiz and Sabanis 2024).

### Bayesian neural network

Similarly to Kuntz et al. (2023); Lim et al. (2024) we also consider a Bayesian Neural Network (BNN) example to perform character classification on the MNIST dataset. In this case our approach is similar to “cold” posterior estimation, as our sampling density is proportional to a density raised to the inverse temperature, as in Wenzel et al. (2020).

This dataset and model provide a more challenging problem for the KIPLMC1 and KIPLMC2 algorithms as the posteriors are known to be multi-modal and hence do not satisfy assumption **H**1. Indeed, we consider this example for illustrative purposes, showing the relative strength of our proposed methods compared to similar interacting particle system (IPS) methods in a non-convex setting. MNIST contains 70’000 28×28 grey-scale images $\{f_i\}_{i=1}^{70000} \subset \mathbb {R}^{784}$. However, to avoid issues of high dimensionality, we consider a normalised subset of 1,000 characters containing only images of fours and nines, whose similarity should pose a challenge. Due to this smaller sample, we are also able to avoid the need for sub-sampling.

Following Kuntz et al. (2023) we employ a two layer BNN with tanh activation function, softmax output layer and 40 dimensional latent space. i.e. we consider the probability of the data labels *l* to be conditionally independent, given the features *f* and the weights x=(w,v), with model,

$$\begin{aligned} p(l|f, x) \propto \exp \left( \sum _{j=1}^{40} v_{lj} \tanh \left( \sum _{i=1}^{784} w_{ji} f_i\right) \right) , \end{aligned}$$

where $w\in \mathbb {R}^{40\times 784}$, $v\in \mathbb {R}^{2\times 40}$. The model has no bias terms and we place the following Gaussian priors on the weights: $w\sim \mathcal {N}(0, e^{2\alpha }\mathbb {I})$ and $v\sim \mathcal {N}(0, e^{2\beta }\mathbb {I})$. Rather than assigning these priors, we learn the parameters $\theta =(\alpha , \beta )\in \mathbb {R}^2$ jointly with the latent variables *x*. Hence, the model’s density is given as

$$\begin{aligned}&p_\theta (x, \{l\}_{i=1}^{1000} |\{f_i\}_{i=1}^{1000})\\&\quad =\mathcal {N}(w;0, e^{2\alpha }) \mathcal {N}(v; 0, e^{2\beta })\prod _{i=1}^{1000} p(l_i|f_i, x). \end{aligned}$$

We will employ autograd methods from the PyTorch library to compute the gradients.

In Fig. 4 we make a comparison between the different algorithms over 100 runs, comparing the log predictive point-wise density (LPPD) and relative error. The LPPD is the average log-likelihood assigned to the correct response, whilst the relative error is given as the accuracy of prediction on a test set (see D.4 for more detail). Observe the improved behaviour of the momentum-based algorithms in LPPD, as well as relative error. We also note the significant acceleration observed for KIPLMC2, highlighting the effect of the distinct numerical integrators. Similarly, in Fig. 4c we can see the more concentrated posterior distributions of the momentum-based algorithms. Thus, it is clear that the KIPLMC1 and KIPLMC2 algorithms perform competitively against other particle-based methods, even in very complex problems, in which our assumptions may not be satisfied.

## Conclusions

This paper extends a line of work on interacting particle-, and more generally, diffusion-based algorithms for maximum marginal likelihood estimation. This paper has focused on alternatives to the IPSs proposed by Kuntz et al. (2023), Akyildiz et al. (2025), Lim et al. (2024) for the MMLE problem by considering an accelerated variant. We have shown that we can leverage the existing literature on underdamped Langevin diffusion for sampling (Dalalyan and Riou-Durand 2020; Cheng et al. 2018; Monmarché 2021; Ma et al. 2021) to produce two algorithms with greater stability, added smoothness and exponential convergence, which concentrate onto the MMLE with quantitative nonasymptotic bounds on the estimation error ${\mathbb {E}}[\Vert \theta _n - \bar{\theta }_\star \Vert ^2]^{1/2}$. In particular, we show that KIPLMC1 attains an accelerated error rate for error ε after $\widetilde{\mathcal {O}}(\sqrt{d_x} \varepsilon ^{-1})$ steps, compared to the IPLA and PGD which require $\widetilde{\mathcal {O}}(d_x \varepsilon ^{-2})$ steps. The proposed algorithms both perform well empirically compared to the MPGD and other particle methods. Specifically, the induced noise should improve guarantees in the non-convex setting, as discussed in Akyildiz et al. (2025), Raginsky et al. (2017), Akyildiz and Sabanis (2024).

The results presented here hold under assumptions of gradient-Lipschitzness and strong log-concavity, however, it is possible to extend this line of work to the non-convex, super-linear or even proximal cases (for examples of this see, Zhang et al. 2023; Akyildiz and Sabanis 2024; Johnston et al. 2024; Cordero-Encinar et al. 2025). Another interesting direction is to consider the setting in Fan et al. (2023), using an accelerated algorithm for high-dimensional linear models. Our methods can also be adapted to solving linear inverse problems as done by Akyildiz et al. (2022), Glyn-Davies et al. (2025). Similar ideas can be considered within the setting of Stein variational gradient descent as done in Sharrock et al. (2024). Crucially, the procedure used to obtain the bounds for the algorithms presented here can easily be extended to other cases, creating a powerful theoretical framework for further underdamped Langevin sampling algorithms for the MMLE problem.

## Figures and Tables

**Fig. 5:**
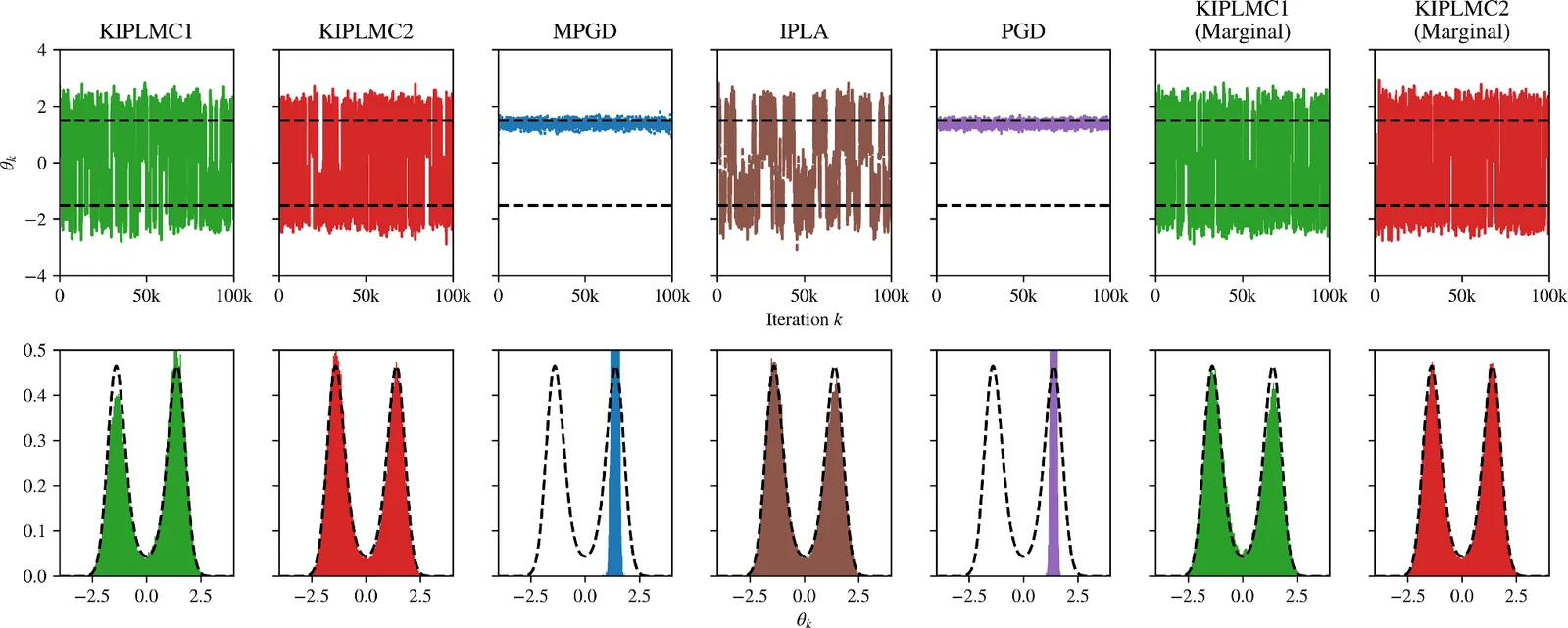
Multi-well Experiment. The trace plots for KIPLMC1, KIPLMC2, MPGD, IPLA and PGD on the multi-well experiment, together with density plots. The black dashed lines in the trace plots indicate the observationally equivalent data-generating parameter values $\theta =\pm 1.5$, while the black dashed curves in the density plots show the target θ-density proportional to $p_\theta (y)^N$. N=10, whilst the friction and step-size are optimised for each algorithm

**Fig. 6:**
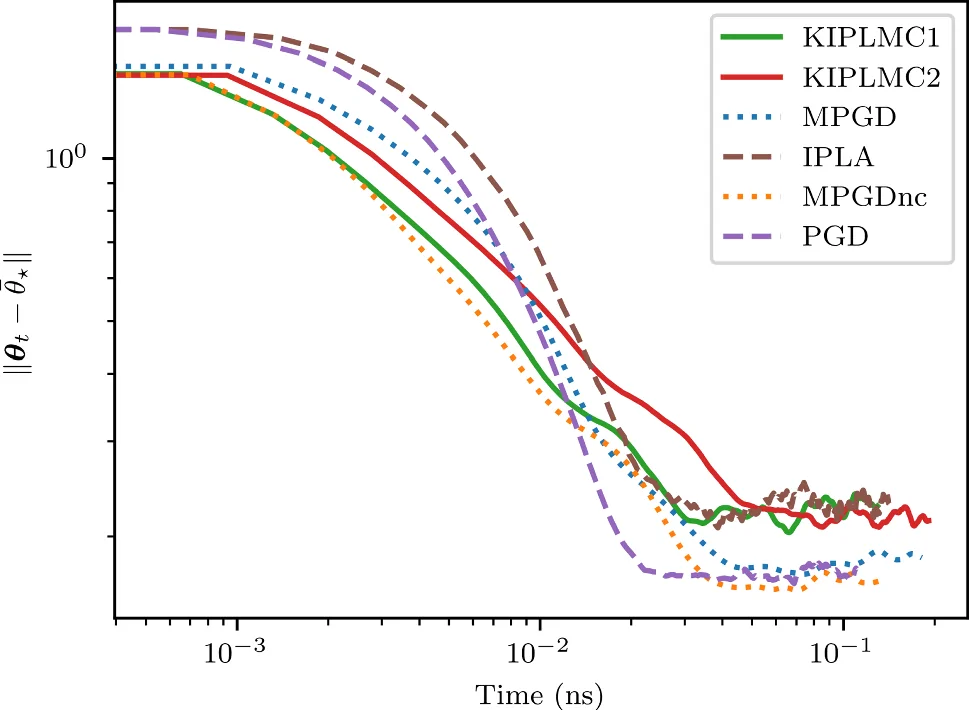
Runtime Comparison. We compare the MSE for the synthetic logistic regression experiment performed in Sect. 5.1 with the same hyperparameters $d_x=d_\theta =3$, $d_y=100$, γ=2.2 and N=100. The results shown are the average of 100 Monte Carlo simulations

## Data Availability

No datasets were generated during the current study and only publicly available datasets were used.

## Preliminary results

In this section, we prove a key result that rewrites the KIPLD as a single underdamped Langevin diffusion evolving in ${\mathbb {R}}^{d_\theta + N d_x}$ with a rescaled potential function and friction coefficient. This will be useful in the analysis of the KIPLD and its discretisation, as it allows us to leverage existing results on underdamped Langevin diffusions.

### Proposition A.1

Let

$$\begin{aligned} ({\boldsymbol{\theta }}_t,{{\textbf{X}}}_t^1,\ldots ,{{\textbf{X}}}_t^N,{{\textbf{V}}}_t^\theta ,{{\textbf{V}}}_t^{x_1},\ldots ,{{\textbf{V}}}_t^{x_N})_{t\ge 0} \end{aligned}$$

solve (KIPLD). Define

$$\begin{aligned} \widetilde{{\boldsymbol{\theta }}}_t&:= {\boldsymbol{\theta }}_{\sqrt{N}t}, \qquad \widetilde{{{\textbf{X}}}}_t^i := N^{-1/2}{{\textbf{X}}}^i_{\sqrt{N}t}, \\ \widetilde{{{\textbf{V}}}}_t^\theta&:= \sqrt{N}{{\textbf{V}}}^\theta _{\sqrt{N}t}, \qquad \widetilde{{{\textbf{V}}}}_t^{x_i} := {{\textbf{V}}}^{x_i}_{\sqrt{N}t}, \end{aligned}$$

for i∈[N], and set

$$\begin{aligned} \widetilde{{{\textbf{Z}}}}_t&:= (\widetilde{{\boldsymbol{\theta }}}_t,\widetilde{{{\textbf{X}}}}_t^1,\ldots ,\widetilde{{{\textbf{X}}}}_t^N)^\intercal \in {\mathbb {R}}^{d_\theta +Nd_x}, \\ \widetilde{{{\textbf{V}}}}_t^z&:= (\widetilde{{{\textbf{V}}}}_t^\theta ,\widetilde{{{\textbf{V}}}}_t^{x_1},\ldots ,\widetilde{{{\textbf{V}}}}_t^{x_N})^\intercal \in {\mathbb {R}}^{d_\theta +Nd_x}, \end{aligned}$$

and

A1 $$\begin{aligned} \bar{U}_N(\theta ,x_1,\ldots ,x_N)&:= \frac{1}{N}\sum _{i=1}^N U(\theta ,\sqrt{N}\,x_i), \end{aligned}$$

and $\widetilde{\gamma }:= \sqrt{N}\gamma$. Then there exists a ${\mathbb {R}}^{d_\theta +Nd_x}$-valued Brownian motion $(\widetilde{{{\textbf{B}}}}_t)_{t\ge 0}$ such that

A2 $$\begin{aligned} {\textrm{d}}\widetilde{{{\textbf{Z}}}}_t&= \widetilde{{{\textbf{V}}}}_t^z\,{\textrm{d}}t, \nonumber \\ {\textrm{d}}\widetilde{{{\textbf{V}}}}_t^z&= -\widetilde{\gamma }\,\widetilde{{{\textbf{V}}}}_t^z\,{\textrm{d}}t - N\nabla _z \bar{U}_N(\widetilde{{{\textbf{Z}}}}_t)\,{\textrm{d}}t + \sqrt{2\widetilde{\gamma }}\,{\textrm{d}}\widetilde{{{\textbf{B}}}}_t. \end{aligned}$$

### Proof

By definition of the rescaled variables, we have

$$\begin{aligned} {\textrm{d}}\widetilde{{\boldsymbol{\theta }}}_t = {\textrm{d}}{\boldsymbol{\theta }}_{s(t)} = {{\textbf{V}}}^\theta _{s(t)}\,{\textrm{d}}s(t) = \sqrt{N}\,{{\textbf{V}}}^\theta _{\sqrt{N} t}\,{\textrm{d}}t = {\widetilde{{{\textbf{V}}}}}^\theta _t\,{\textrm{d}}t \end{aligned}$$

where $s(t):=\sqrt{N}t$. From (KIPLD), ${\textrm{d}}{{\textbf{X}}}_t^i={{\textbf{V}}}_t^{x_i}{\textrm{d}}t$, so

$$\begin{aligned} {\textrm{d}}{{\textbf{X}}}^i_{s(t)} = {{\textbf{V}}}^{x_i}_{s(t)}{\textrm{d}}s(t) = \sqrt{N}\,{{\textbf{V}}}^{x_i}_{\sqrt{N}t}{\textrm{d}}t. \end{aligned}$$

Hence

$$\begin{aligned} {\textrm{d}}\widetilde{{{\textbf{X}}}}_t^i = N^{-1/2}\,{\textrm{d}}{{\textbf{X}}}^i_{\sqrt{N}t} = N^{-1/2}\,\sqrt{N}\,{{\textbf{V}}}^{x_i}_{\sqrt{N}t}{\textrm{d}}t = \widetilde{{{\textbf{V}}}}_t^{x_i}{\textrm{d}}t, \end{aligned}$$

and therefore ${\textrm{d}}\widetilde{{{\textbf{Z}}}}_t=\widetilde{{{\textbf{V}}}}_t^z{\textrm{d}}t$. Next, define $\widetilde{{{\textbf{B}}}}_t^j:=N^{-1/4}{{\textbf{B}}}_{\sqrt{N}t}^j$ for j=0,…,N; then each $\widetilde{{{\textbf{B}}}}_t^j$ is a Brownian motion and ${\textrm{d}}{{\textbf{B}}}_{\sqrt{N}t}^j = N^{1/4}{\textrm{d}}\widetilde{{{\textbf{B}}}}_t^j.$ Using (KIPLD), set $s(t):=\sqrt{N}t$. Then

$$\begin{aligned}&{\textrm{d}}\widetilde{{{\textbf{V}}}}_t^\theta = \sqrt{N}\,{\textrm{d}}{{\textbf{V}}}^\theta _{s(t)}\\&= \sqrt{N}\left[ \left( -\gamma {{\textbf{V}}}^\theta _{s(t)} - \frac{1}{N}\!\sum _{i=1}^N \nabla _\theta U({\boldsymbol{\theta }}_{s(t)},{{\textbf{X}}}^i_{s(t)})\right) {\textrm{d}}s(t)+ \sqrt{\frac{2\gamma }{N}}\,{\textrm{d}}{{\textbf{B}}}^0_{s(t)} \right] \\&= -N\gamma {{\textbf{V}}}^\theta _{\sqrt{N}t}{\textrm{d}}t - \sum _{i=1}^N \nabla _\theta U({\boldsymbol{\theta }}_{\sqrt{N}t},{{\textbf{X}}}^i_{\sqrt{N}t}){\textrm{d}}t + \sqrt{2\gamma \sqrt{N}}\,{\textrm{d}}\widetilde{{{\textbf{B}}}}_t^0\\&= -\widetilde{\gamma }\,\widetilde{{{\textbf{V}}}}_t^\theta {\textrm{d}}t - N\nabla _\theta \bar{U}_N(\widetilde{{{\textbf{Z}}}}_t){\textrm{d}}t + \sqrt{2\widetilde{\gamma }}\,{\textrm{d}}\widetilde{{{\textbf{B}}}}_t^0, \end{aligned}$$

where we used ${\textrm{d}}s(t)=\sqrt{N}{\textrm{d}}t$ and

$$ N\nabla _\theta \bar{U}_N(\widetilde{{{\textbf{Z}}}}_t) = \sum _{i=1}^N \nabla _\theta U({\boldsymbol{\theta }}_{\sqrt{N}t},{{\textbf{X}}}^i_{\sqrt{N}t}). $$

Fix i∈[N]. Using again $s(t)=\sqrt{N}t$ and (KIPLD),

$$\begin{aligned} {\textrm{d}}\widetilde{V}_t^{x_i}&= {\textrm{d}}{{\textbf{V}}}^{x_i}_{s(t)}\\&= \left( -\gamma {{\textbf{V}}}^{x_i}_{s(t)} - \nabla _x U({\boldsymbol{\theta }}_{s(t)},{{\textbf{X}}}^i_{s(t)})\right) {\textrm{d}}s(t) + \sqrt{2\gamma }\,{\textrm{d}}{{\textbf{B}}}^i_{s(t)} \\&= -\sqrt{N}\gamma {{\textbf{V}}}^{x_i}_{\sqrt{N}t}{\textrm{d}}t - \sqrt{N}\,\nabla _x U({\boldsymbol{\theta }}_{\sqrt{N}t},{{\textbf{X}}}^i_{\sqrt{N}t}){\textrm{d}}t + \sqrt{2\gamma \sqrt{N}}\,{\textrm{d}}\widetilde{{{\textbf{B}}}}_t^i\\&= -\widetilde{\gamma }\,\widetilde{{{\textbf{V}}}}_t^{x_i}{\textrm{d}}t - N\nabla _{z_i}\bar{U}_N(\widetilde{{{\textbf{Z}}}}_t){\textrm{d}}t + \sqrt{2\widetilde{\gamma }}\,{\textrm{d}}\widetilde{{{\textbf{B}}}}_t^i, \end{aligned}$$

where

$$ N\nabla _{z_i}\bar{U}_N(\widetilde{{{\textbf{Z}}}}_t) = \sqrt{N}\,\nabla _x U({\boldsymbol{\theta }}_{\sqrt{N}t},{{\textbf{X}}}^i_{\sqrt{N}t}). $$

Stacking coordinates and defining $\widetilde{{{\textbf{B}}}}_t = (\widetilde{{{\textbf{B}}}}_t^0,\widetilde{{{\textbf{B}}}}_t^1,\ldots ,\widetilde{{{\textbf{B}}}}_t^N)^\intercal$ gives (A2). □

Next we show that the stationary measure of the dynamics in (A2) is given by a Gibbs measure with potential function $N\bar{U}_N$.

### Lemma A.1

(Stationary measure for (A2)) The measure $\tilde{\pi }$ invariant for the dynamics in (A2) is given as

A3 $$\begin{aligned} \tilde{\pi }({\textrm{d}}\tilde{z}, {\textrm{d}}\tilde{v}) \propto \exp \left( -N\bar{U}_N(\tilde{z}) -\frac{1}{2}\Vert \tilde{v}\Vert ^2\right) {\textrm{d}}\tilde{z} {\textrm{d}}\tilde{v}, \end{aligned}$$

for all $\tilde{z},\tilde{v}\in \mathbb {R}^{d_z}$, where $d_z=d_\theta +Nd_x$.

This result follows directly by observing the rescaling given in Proposition A.1 and the standard form of the stationary measure for underdamped Langevin diffusions. We now show that $\bar{U}_N$ is μ-strongly convex under **H**1.

### Lemma A.2

(Strong convexity of $\bar{U}_N$) Under **H**1, the function $\bar{U}_N: {\mathbb {R}}^{d_\theta + Nd_x} \rightarrow {\mathbb {R}}$ as defined in (A1) is μ-strongly convex

$$\begin{aligned} \langle z-z^\prime , \nabla \bar{U}_N(z) - \nabla \bar{U}_N(z^\prime )\rangle \ge \mu \Vert z-z^\prime \Vert ^2, \end{aligned}$$

for all $z, z^\prime \in {\mathbb {R}}^{d_\theta + Nd_x}$ and $N \in {\mathbb {N}}$.

### Proof

Let

$$ z=(z_\theta ,z_1,\dots ,z_N),\qquad z'=(z_\theta ',z_1',\dots ,z_N'). $$

From

$$ \bar{U}_N(z)=\frac{1}{N}\sum _{i=1}^N U(z_\theta ,\sqrt{N}\,z_i), $$

we have

$$\begin{aligned} \nabla _{z_\theta }\bar{U}_N(z)&=\frac{1}{N}\sum _{i=1}^N \nabla _\theta U(z_\theta ,\sqrt{N} z_i), \\ \nabla _{z_i}\bar{U}_N(z)&= \frac{1}{\sqrt{N}}\nabla _x U(z_\theta ,\sqrt{N} z_i). \end{aligned}$$

Hence

$$\begin{aligned} \langle&z-z', \nabla \bar{U}_N(z)-\nabla \bar{U}_N(z')\rangle \\&=\frac{1}{N}\sum _{i=1}^N \langle z_\theta -z_\theta ',\Delta _{\theta ,i}\rangle +\frac{1}{\sqrt{N}}\sum _{i=1}^N \langle z_i-z_i',\Delta _{x,i}\rangle , \end{aligned}$$

where

$$ \Delta _{\theta ,i}:=\nabla _\theta U(z_\theta ,\sqrt{N} z_i)-\nabla _\theta U(z_\theta ',\sqrt{N} z_i'), $$

$$ \Delta _{x,i}:=\nabla _x U(z_\theta ,\sqrt{N} z_i)-\nabla _x U(z_\theta ',\sqrt{N} z_i'). $$

Now apply Assumption **H**1 to the two points

$$ (z_\theta ,\sqrt{N} z_i),\qquad (z_\theta ',\sqrt{N} z_i'). $$

For each i,

$$\begin{aligned} \langle z_\theta -z_\theta ',\Delta _{\theta ,i}\rangle&+\sqrt{N}\,\langle z_i-z_i',\Delta _{x,i}\rangle \\ &\ge \mu \big (\Vert z_\theta -z_\theta '\Vert ^2+N\Vert z_i-z_i'\Vert ^2\big ). \end{aligned}$$

Divide by N and sum over i:

$$\begin{aligned} \langle z-z',&\nabla \bar{U}_N(z)- \nabla \bar{U}_N(z')\rangle \\&\ge \mu \Vert z_\theta -z_\theta '\Vert ^2+\mu \sum _{i=1}^N\Vert z_i-z_i'\Vert ^2 =\mu \Vert z-z'\Vert ^2. \end{aligned}$$

So $\bar{U}_N$ is μ-strongly convex. □

Next, we show that the function $\bar{U}_N$ is *L*-gradient Lipschitz.

### Lemma A.3

(Gradient Lipschitzness of $\bar{U}_N$) Under **H**2, the function $\bar{U}_N: {\mathbb {R}}^{d_\theta + Nd_x} \rightarrow {\mathbb {R}}$ as defined in (A1) is *L*-gradient Lipschitz

$$\begin{aligned} \Vert \nabla \bar{U}_N(z) - \nabla \bar{U}_N(z^\prime )\Vert \le L \Vert z-z^\prime \Vert , \end{aligned}$$

for all $z, z^\prime \in {\mathbb {R}}^{d_\theta + Nd_x}$ and $N \in {\mathbb {N}}$.

### Proof

Let $z = (z_\theta , z_1, \ldots , z_N)$ and $z^\prime = (z_\theta ^\prime , z_1^\prime , \ldots , z_N^\prime )$. Then

$$\begin{aligned} \Vert&\nabla \bar{U}_N(z) - \nabla \bar{U}_N(z^\prime )\Vert ^2 \\&= \left\| \frac{1}{N} \sum _{i=1}^N \left( \nabla _\theta U(z_\theta , \sqrt{N}z_i) - \nabla _\theta U(z_\theta ^\prime , \sqrt{N}z_i^\prime )\right) \right\| ^2\\&\quad + \frac{1}{N} \sum _{i=1}^N \left\| \nabla _x U(z_\theta , \sqrt{N}z_i) - \nabla _x U(z_\theta ^\prime , \sqrt{N}z_i^\prime )\right\| ^2. \end{aligned}$$

Using the fact that $\Vert \cdot \Vert ^2$ is a convex function and Jensen’s inequality for the first term, we obtain

$$\begin{aligned} \Vert \nabla&\bar{U}_N(z) - \nabla \bar{U}_N(z^\prime )\Vert ^2 \\ &\le \frac{1}{N} \sum _{i=1}^N \left\| \nabla _\theta U(z_\theta , \sqrt{N}z_i) - \nabla _\theta U(z_\theta ^\prime , \sqrt{N}z_i^\prime )\right\| ^2\\&+ \frac{1}{N} \sum _{i=1}^N \left\| \nabla _x U(z_\theta , \sqrt{N}z_i) - \nabla _x U(z_\theta ^\prime , \sqrt{N}z_i^\prime )\right\| ^2. \end{aligned}$$

Using **H**2, we have

$$\begin{aligned} \Vert \nabla \bar{U}_N(z)&- \nabla \bar{U}_N(z^\prime )\Vert ^2 \\&\le \frac{L^2}{N} \sum _{i=1}^N \left( \Vert z_\theta - z_\theta ^\prime \Vert ^2 + N \Vert z_i - z_i^\prime \Vert ^2\right) \\&= L^2 \Vert z - z^\prime \Vert ^2, \end{aligned}$$

which completes the proof. □

## Equivalence of algorithms

We prove in Proposition A.1 that the KIPLD can be rewritten as a single underdamped Langevin diffusion evolving in ${\mathbb {R}}^{d_\theta + N d_x}$. This brings the natural question about the algorithms we derived, namely, KIPLMC1 and KIPLMC2, which are discretisations of the KIPLD, and how they relate to the standard discretisation schemes the rescaled diffusion in (A2). We show below that the standard discretisations of (A2) are precisely the KIPLMC1 and KIPLMC2 algorithms we defined in Sect. 3. This will later allow us to leverage existing results on the convergence of these standard discretisation schemes to obtain convergence results for our algorithms.

### KIPLMC1

We show below the exponential integrator algorithm, presented in Dalalyan and Riou-Durand (2020), for our diffusion (KIPLD) matches the KIPLMC1 algorithm we defined in Sect. 3.

#### Proposition B.1

Let the sequence $(\widetilde{Z}_k,\widetilde{V}_k^z)_{k\ge 0}$ be the exponential-integrator discretisation of the rescaled underdamped diffusion (A2) with friction parameter $\widetilde{\gamma }=\sqrt{N}\gamma$ and step-size $\widetilde{\eta }=\eta /\sqrt{N}$. Define the unscaled variables by

$$\begin{aligned} \widetilde{\theta }_k&= \theta _k,\qquad \widetilde{X}_k^i = N^{-1/2}X_k^i,\\ \widetilde{V}_k^\theta&= \sqrt{N}V_k^\theta ,\qquad \widetilde{V}_k^{x_i} = V_k^{x_i}, \end{aligned}$$

for each i∈[N]. Then, after the corresponding rescaling of the Gaussian noise variables, the resulting recursion in $(\theta _k,X_k^i,V_k^\theta ,V_k^{x_i})_{i\in [N]}$ coincides exactly with the KIPLMC1 scheme defined in Sect. 3.1.

#### Proof

Let us recall that, by Proposition A.1, we can rewrite (KIPLD) as the standard underdamped SDE

$$\begin{aligned} {\textrm{d}}\widetilde{{{\textbf{Z}}}}_t&= \widetilde{{{\textbf{V}}}}_t^z\,{\textrm{d}}t, \\ {\textrm{d}}\widetilde{{{\textbf{V}}}}_t^z&= -\widetilde{\gamma }\,\widetilde{{{\textbf{V}}}}_t^z\,{\textrm{d}}t - N\nabla _z \bar{U}_N(\widetilde{{{\textbf{Z}}}}_t)\,{\textrm{d}}t + \sqrt{2\widetilde{\gamma }}\,{\textrm{d}}\widetilde{{{\textbf{B}}}}_t, \end{aligned}$$

where $\tilde{\gamma } = \sqrt{N} \gamma$ is the friction parameter and we also implicitly adjust its initial condition to be rescaled. We discretise this diffusion using the exponential integrator scheme (termed KLMC1) developed by Dalalyan and Riou-Durand (2020). In order to do this, define a sequence of functions $\tilde{\psi }_{k+1}^t = \int _0^t \tilde{\psi }_k^s {\textrm{d}}s$ where $\tilde{\psi }_0^t = e^{-\tilde{\gamma } t}$. With this definition, the exponential integrator scheme with step-size $\tilde{\eta }$ is defined as

$$\begin{aligned} \widetilde{Z}_{k+1}&= \widetilde{Z}_k + \tilde{\psi }_1^{\tilde{\eta }} \tilde{V}_k - \tilde{\psi }_2^{\tilde{\eta }} N\nabla _z \bar{U}_N(\widetilde{Z}_k) + \sqrt{2\tilde{\gamma }}\zeta _{k+1}^\prime \\ \widetilde{V}_{k+1}&= \tilde{\psi }_0^{\tilde{\eta }} \widetilde{V}_k - \tilde{\psi }_1^{\tilde{\eta }} N \nabla _z \bar{U}_N(\tilde{Z}_k) + \sqrt{2\tilde{\gamma }} \zeta _{k+1}, \end{aligned}$$

where the stacked vector $$[(\zeta _k)_1, \dots , (\zeta _k)_{d_z},(\zeta '_k)_1, \dots $$$$(\zeta '_k)_{d_z}]^\top $$ is a centred Gaussian random variable with covariance $$\widetilde{C}\otimes I_{d_z}$$, where $$\widetilde{C}$$ is given by

$$\begin{aligned} \widetilde{C} = \int _0^{\tilde{\eta }} \begin{bmatrix} \tilde{\psi }_0^t \\ \tilde{\psi }_1^t \end{bmatrix} \begin{bmatrix} \tilde{\psi }_0^t&\tilde{\psi }_1^t \end{bmatrix} \textrm{d}t. \end{aligned}$$

Written explicitly via the definition of $\tilde{\psi }_i^t$, we have,

$$\begin{aligned} \widetilde{C}= \frac{1}{2\tilde{\gamma }} \begin{pmatrix} (1-e^{-2\tilde{\gamma }\tilde{\eta }}) & \frac{(e^{-\tilde{\gamma }\tilde{\eta }} -1)^2}{\tilde{\gamma }} \\ \frac{(e^{-\tilde{\gamma }\tilde{\eta }} -1)^2}{\tilde{\gamma }} & \frac{2\tilde{\gamma }\tilde{\eta } + 4 e^{-\tilde{\gamma }\tilde{\eta }}- e^{-2\tilde{\gamma }\tilde{\eta }} -3}{\tilde{\gamma }^2} \end{pmatrix} \end{aligned}$$

Let us now relate the matrix $\widetilde{C}$ to the matrix we have defined in (7) for the KIPLMC1 scheme. Written explicitly with the definitions in Sect. 3.1, KIPLMC1 instead defines functions $\psi _0^\eta = e^{-\gamma \eta }$ and $\psi _{i+1}^\eta = \int _0^\eta \psi _i^t {\textrm{d}}t$, and the covariance matrix in (7) is given as

$$\begin{aligned} C=\frac{1}{2\gamma } \begin{pmatrix} (1-e^{- 2 \gamma \eta }) & \frac{(e^{- \gamma \eta }-1)^2}{\gamma }\\ \frac{(e^{-\gamma \eta }-1)^2}{\gamma } & \frac{2\gamma \eta + 4e^{-\gamma \eta } - e^{-2\gamma \eta }-3}{\gamma ^2} \end{pmatrix}. \end{aligned}$$

Recall that the stacked vector $[(\varepsilon _k)_1, (\varepsilon '_k)_1, (\varepsilon _k)_2, (\varepsilon '_k)_2, \dots ,$ $((\varepsilon _k)_{d_z}, (\varepsilon '_k)_{d_z})]^\top$ is a centred Gaussian random variable with covariance $C\otimes I_{d_z}$ as defined in Sect. 3.1. Note the relation between $\widetilde{C}$ and *C* is given by

$$\begin{aligned} \widetilde{C}= \begin{pmatrix} N^{-\frac{1}{2}} C_{11} & N^{-1} C_{12}\\ N^{-1} C_{21} & N^{-\frac{3}{2}} C_{22} \end{pmatrix}. \end{aligned}$$

Hence, we recover

$$\begin{aligned} \zeta _{k}&= (N)^{-\frac{1}{4}} (\varepsilon _k^0, \dots , \varepsilon _k^N)^\top ,\\ \zeta _k^\prime&= (N)^{-\frac{3}{4}} (\varepsilon ^{0,\prime }_k, \dots , \varepsilon _k^{N,\prime })^\top . \end{aligned}$$

We first write the full exponential-integrator scheme coordinatewise, without yet substituting the relations between $\tilde{\eta }$ and η or between the rescaled and original variables. This gives

$$\begin{aligned} \widetilde{\theta }_{k+1}&= \widetilde{\theta }_k + \tilde{\psi }_1^{\tilde{\eta }} \widetilde{V}^\theta _k - \tilde{\psi }_2^{\tilde{\eta }} N \nabla _\theta \bar{U}_N(\widetilde{Z}_k) + \sqrt{2\tilde{\gamma }}(\zeta _{k}^\prime )^0,\\ \widetilde{X}_{k+1}^i&= \widetilde{X}_k^i + \tilde{\psi }_1^{\tilde{\eta }} \widetilde{V}_k^{x_i} - \tilde{\psi }_2^{\tilde{\eta }} N \nabla _{z_i} \bar{U}_N(\widetilde{Z}_k) + \sqrt{2\tilde{\gamma }}(\zeta _{k}^\prime )^i,\\ \widetilde{V}_{k+1}^\theta&= \tilde{\psi }_0^{\tilde{\eta }} \widetilde{V}_k^\theta - \tilde{\psi }_1^{\tilde{\eta }} N \nabla _\theta \bar{U}_N(\widetilde{Z}_k) + \sqrt{2\tilde{\gamma }}(\zeta _{k})^0,\\ \widetilde{V}_{k+1}^{x_i}&= \tilde{\psi }_0^{\tilde{\eta }} \widetilde{V}_k^{x_i} - \tilde{\psi }_1^{\tilde{\eta }} N \nabla _{z_i} \bar{U}_N(\widetilde{Z}_k) + \sqrt{2\tilde{\gamma }}(\zeta _{k})^i, \end{aligned}$$

where the noise variables $(\zeta _k)^0$, $(\zeta _k^\prime )^0$, $(\zeta _k)^i$ and $(\zeta _k^\prime )^i$ are the appropriate coordinates of $\zeta _k$ and $\zeta _k^\prime$ respectively. Now recall from Proposition A.1 that $\tilde{\gamma } = \sqrt{N}\gamma$, and choose $\tilde{\eta }=\eta /\sqrt{N}$. Then

$$\begin{aligned} \tilde{\psi }_0^{\tilde{\eta }}&= e^{-\gamma \eta } = \psi _0^\eta ,\\ \tilde{\psi }_1^{\tilde{\eta }}&= \frac{1}{\gamma \sqrt{N}} (1-e^{-\gamma \eta }) = \frac{\psi _1^\eta }{\sqrt{N}},\\ \tilde{\psi }_2^{\tilde{\eta }}&= \frac{1}{\gamma N}(\eta - \frac{1}{\gamma }(1-e^{-\gamma \eta })) = \frac{\psi _2^\eta }{N}. \end{aligned}$$

Using also the identities

B4 $$\begin{aligned} \begin{aligned} N\nabla _\theta \bar{U}_N(\widetilde{Z}_k)&= \sum _{i=1}^N \nabla _\theta U(\widetilde{\theta }_k,\sqrt{N}\widetilde{X}_k^i),\\ N\nabla _{z_i} \bar{U}_N(\widetilde{Z}_k)&= \sqrt{N}\nabla _x U(\widetilde{\theta }_k,\sqrt{N}\widetilde{X}_k^i), \end{aligned} \end{aligned}$$

together with $\widetilde{\theta }_k=\theta _k$, $\widetilde{X}_k^i=N^{-1/2}X_k^i$, $\widetilde{V}_k^\theta =\sqrt{N}V_k^\theta$, $\widetilde{V}_k^{x_i}=V_k^{x_i}$, $(\zeta _k)^0=N^{-1/4}\varepsilon _k^0$, $(\zeta _k^\prime )^0=N^{-3/4}\varepsilon _k^{0,\prime }$, $(\zeta _k)^i=N^{-1/4}\varepsilon _k^i$, and $(\zeta _k^\prime )^i=N^{-3/4}\varepsilon _k^{i,\prime }$, we obtain for the θ-coordinates

$$\begin{aligned} \theta _{k+1}&= \theta _k + \frac{\psi _1^\eta }{\sqrt{N}}\widetilde{V}^\theta _k - \frac{\psi _2^\eta }{N} \sum _{i=1}^N \nabla _\theta U(\theta _k, X_k^i)+ \sqrt{\frac{2\gamma }{N}} \varepsilon ^{0,\prime }_{k},\\ \widetilde{V}^\theta _{k+1}&= \psi _0^\eta \widetilde{V}^\theta _k - \frac{\psi _1^\eta }{\sqrt{N}} \sum _{i=1}^N \nabla _\theta U(\theta _k, X_k^i) + \sqrt{2\gamma } \varepsilon ^0_{k}. \end{aligned}$$

Since $\widetilde{V}_k^\theta =\sqrt{N}V_k^\theta$, substituting this relation into the first line and dividing the second line by $\sqrt{N}$ yields

$$\begin{aligned} \theta _{k+1}&= \theta _k + \psi _1^\eta V^\theta _k - \frac{\psi _2^\eta }{N} \sum _{i=1}^N \nabla _\theta U(\theta _k, X_k^i)+ \sqrt{\frac{2\gamma }{N}} \varepsilon ^{0,\prime }_{k},\\ V^\theta _{k+1}&= \psi _0^\eta V^\theta _k - \frac{\psi _1^\eta }{N} \sum _{i=1}^N \nabla _\theta U(\theta _k, X_k^i) + \sqrt{\frac{2\gamma }{N}} \varepsilon ^0_{k}. \end{aligned}$$

For each particle coordinate i∈[N], the same substitutions give

$$\begin{aligned} \widetilde{X}_{k+1}^i&= \widetilde{X}_k^i + \frac{\psi _1^\eta }{\sqrt{N}}V^{x_i}_k - \frac{\psi _2^\eta }{\sqrt{N}} \nabla _x U(\theta _k, X_k^i)+ \sqrt{\frac{2\gamma }{N}} \varepsilon ^{i,\prime }_{k},\\ V^{x_i}_{k+1}&= \psi _0^\eta V^{x_i}_k - \psi _1^\eta \nabla _x U(\theta _k, X_k^i) + \sqrt{2\gamma } \varepsilon ^i_{k}. \end{aligned}$$

Finally, multiplying the first equation by $\sqrt{N}$, i.e. using $X_k^i=\sqrt{N}\widetilde{X}_k^i$, yields

$$\begin{aligned} X^i_{k+1}&= X^i_k + \psi _1^\eta V^{x_i}_k - \psi _2^\eta \nabla _x U(\theta _k, X_k^i)+ \sqrt{2\gamma } \varepsilon ^{i,\prime }_{k}\\ V^{x_i}_{k+1}&= \psi _0^\eta V^{x_i}_k - \psi _1^\eta \nabla _x U(\theta _k, X_k^i) + \sqrt{2\gamma } \varepsilon ^i_{k}. \end{aligned}$$

Together with the θ-update above, this is precisely the KIPLMC1 scheme. □

### KIPLMC2

#### Proposition B.2

Let $(\widetilde{Z}_k,\widetilde{V}_k^z)_{k\ge 0}$ be the OBABO discretisation of the rescaled underdamped diffusion (A2) with friction parameter $\widetilde{\gamma }=\sqrt{N}\gamma$ and step-size $\widetilde{\eta }=\eta /\sqrt{N}$. Define the unscaled variables by

$$\begin{aligned} \widetilde{\theta }_k&= \theta _k,\qquad \widetilde{X}_k^i = N^{-1/2}X_k^i,\\ \widetilde{V}_k^\theta&= \sqrt{N}V_k^\theta ,\qquad \widetilde{V}_k^{x_i} = V_k^{x_i}, \end{aligned}$$

for each i∈[N]. Then the resulting recursion in $$(\theta _k,X_k^i,V_k^\theta ,$$$$V_k^{x_i})_{i\in [N]}$$ coincides exactly with the KIPLMC2 scheme defined in Algorithm 2.

#### Proof

In this case we again apply Proposition A.1 to apply the OBABO results from Monmarché (2021) for the standard underdamped diffusion, to our own. Let us recall the standard form, given in Proposition A.1 with $\tilde{\gamma }=\sqrt{N}\gamma$,

$$\begin{aligned} {\textrm{d}}\widetilde{{{\textbf{Z}}}}_t&= \widetilde{{{\textbf{V}}}}_t^z\,{\textrm{d}}t, \\ {\textrm{d}}\widetilde{{{\textbf{V}}}}_t^z&= -\widetilde{\gamma }\,\widetilde{{{\textbf{V}}}}_t^z\,{\textrm{d}}t - N\nabla _z \bar{U}_N(\widetilde{{{\textbf{Z}}}}_t)\,{\textrm{d}}t + \sqrt{2\widetilde{\gamma }}\,{\textrm{d}}\widetilde{{{\textbf{B}}}}_t, \end{aligned}$$

This diffusion is discretised with the OBABO scheme with step-size $\tilde{\eta }$, to obtain,

$$\begin{aligned} \widetilde{Z}_{k+1}&= \widetilde{Z}_k + \tilde{\eta } \left( \tilde{\delta }\widetilde{V}_k + \sqrt{1-\tilde{\delta }^2}\xi _{k+1}\right) \\&\quad -\frac{\tilde{\eta }^2}{2} N\nabla _z\bar{U}_N(\widetilde{Z}_k)\\ \widetilde{V}_{k+1}&= \tilde{\delta }^2 \widetilde{V}_{k} + \sqrt{1-\tilde{\delta }^2} (\tilde{\delta }\xi _{k+1} + \xi ^\prime _{k+1})\\&\quad -\frac{\tilde{\eta }\tilde{\delta }}{2} N (\nabla _z \bar{U}_N(\widetilde{Z}_k) + \nabla _z \bar{U}_N(\widetilde{Z}_{k+1})), \end{aligned}$$

where we recall that $\tilde{\delta }=e^{-\tilde{\gamma }\tilde{\eta }/2}$. Let us begin at looking at the discretisation coordinatewise in the scaled case,

$$\begin{aligned} \widetilde{\theta }_{k+1}&= \widetilde{\theta }_k + \tilde{\eta }\left( \tilde{\delta } \widetilde{V}^\theta _{k} + \sqrt{1-\tilde{\delta }^2} \xi ^0_{k+1}\right) \\&\quad - \frac{\tilde{\eta }^2}{2} N \nabla _\theta \bar{U}_N(\widetilde{Z}_k),\\ \widetilde{X}^i_{k+1}&= \widetilde{X}^i_k + \tilde{\eta }\left( \tilde{\delta } \widetilde{V}^{x_i}_{k} + \sqrt{1-\tilde{\delta }^2} \xi ^i_{k+1}\right) \\&\quad - \frac{\tilde{\eta }^2}{2} N \nabla _{z_i} \bar{U}_N(\widetilde{Z}_k),\\ \widetilde{V}^\theta _{k+1}&= \tilde{\delta }^2 \widetilde{V}^\theta _k + \sqrt{1-\tilde{\delta }^2} (\tilde{\delta } \xi ^0_{k+1} + \xi _{k+1}^{0,\prime })\\&\quad - \frac{\tilde{\eta }\tilde{\delta }}{2} N(\nabla _\theta \bar{U}_N(\widetilde{Z}_k) + \nabla _\theta \bar{U}_N(\widetilde{Z}_{k+1}),\\ \widetilde{V}^{x_i}_{k+1}&= \tilde{\delta }^2 \widetilde{V}^{x_i}_k + \sqrt{1-\tilde{\delta }^2} (\tilde{\delta } \xi ^i_{k+1} + \xi _{k+1}^{i,\prime })\\&\quad - \frac{\tilde{\eta }\tilde{\delta }}{2} N(\nabla _{z_i} \bar{U}_N(\widetilde{Z}_k) + \nabla _{z_i} \bar{U}_N(\widetilde{Z}_{k+1})). \end{aligned}$$

We now proceed to rescaling the algorithm by observing that $\tilde{\delta }=\delta$ and recalling that $\tilde{\psi }_0^{\tilde{\eta }} = \psi _0^\eta$. Using this, $\tilde{\eta } = \eta / \sqrt{N}$ and $\tilde{\gamma } = \sqrt{N}\gamma$ and the identities (B4), we obtain for the θ-coordinates,

$$\begin{aligned} \theta _{k+1}&= \theta _k + \frac{\eta }{\sqrt{N}} \left( \delta \widetilde{V}^\theta _k + \sqrt{1-\delta ^2}\xi _{k+1}\right) \\ &- \frac{\eta ^2}{2N}\sum _{i=1}^N\nabla _\theta U(\theta _k, X_k^i)\\ \widetilde{V}^\theta _{k+1}&= \delta ^2 \widetilde{V}^\theta _k + \sqrt{1-\delta ^2} (\delta \xi _{k+1} + \xi ^\prime _{k+1}) \\ &- \frac{\eta \delta }{2\sqrt{N}}\sum _{i=1}^N \bigg (\nabla _\theta U(\theta _k, X_k^i) + \nabla _\theta U(\theta _{k+1}, X_{k+1}^i)\bigg ). \end{aligned}$$

Substituting in the relationship $\widetilde{V}_k^\theta =\sqrt{N}V_k^\theta$ and $\widetilde{X}_k^i = X_k^i/\sqrt{N}$, we obtain,

$$\begin{aligned} \theta _{k+1}&= \theta _k + \eta \left( \delta V^\theta _k + \sqrt{\frac{1-\delta ^2}{N}}\xi _{k+1}\right) \\ &- \frac{\eta ^2}{2N}\sum _{i=1}^N\nabla _\theta U(\theta _k, X_k^i)\\ V^\theta _{k+1}&= \delta ^2 V^\theta _k + \sqrt{\frac{1-\delta ^2}{N}} (\delta \xi _{k+1} + \xi ^\prime _{k+1}) \\ &- \frac{\eta \delta }{2N}\sum _{i=1}^N \bigg (\nabla _\theta U(\theta _k, X_k^i) + \nabla _\theta U(\theta _{k+1}, X_{k+1}^i)\bigg ). \end{aligned}$$

For the particles i∈[N], the same substitutions give,

$$\begin{aligned} \widetilde{X}^i_{k+1}&= \widetilde{X}^i_k + \frac{\eta }{\sqrt{N}}\left( \delta V^{x_i}_{k} + \sqrt{1-\delta ^2} \xi ^i_{k+1}\right) \\&\quad - \frac{\eta ^2}{2\sqrt{N}} \nabla _{x_i} U(\theta _k , \sqrt{N}\widetilde{X}_k^i),\\ V^{x_i}_{k+1}&= \delta ^2 V^{x_i}_k + \sqrt{1-\delta ^2} (\delta \xi ^i_{k+1} + \xi _{k+1}^{i,\prime })\\&\quad - \frac{\eta \delta }{2} (\nabla _{x_i} U(\theta _k, \sqrt{N}\widetilde{X}^i_k)\\&\quad + \nabla _{x_i} U(\theta _{k+1}, \sqrt{N}\widetilde{X}^i_{k+1})). \end{aligned}$$

Finally, using $X_k^i = \sqrt{N}\widetilde{X}_k^i$, we recover,

$$\begin{aligned} X^i_{k+1}&= X^i_k + \eta \left( \delta V^{x_i}_k + \sqrt{1-\delta ^2}\xi ^i_{k+1}\right) \\ &\quad - \frac{\eta ^2}{2}\nabla _x U(\theta _k, X_k^i)\\ V^{x_i}_{k+1}&= \delta ^2 V^{x_i}_k + \sqrt{1-\delta ^2} (\delta \xi ^i_{k+1} + \xi ^{i,\prime }_{k+1}) \\ &\quad - \frac{\eta \delta }{2} \bigg (\nabla _x U(\theta _k, X_k^i) + \nabla _x U(\theta _{k+1}, X_{k+1}^i)\bigg ). \end{aligned}$$

This shows that the OBABO discretisation of the rescaled diffusion, with step-size $\tilde{\eta } = \eta /\sqrt{N}$, is precisely the KIPLMC2 scheme. □

#### Remark B.1

The results in Propositions A.1–B.2 significantly streamline our proofs, since they allow us to connect KIPLD and our methods KIPLMC1 and KIPLMC2 to the standard underdamped diffusion and its discretisations, for which there is a rich literature of results that we can apply.

## Proofs of main results

### Proof of Proposition 1

Given Proposition A.1, the system (A2) has a stationary measure given by the density

$$\begin{aligned} \tilde{\pi }(z, \bar{v}) \propto \exp \left( -N\bar{U}_N(z) - \frac{1}{2}\Vert \bar{v}\Vert ^2 \right) , \end{aligned}$$

where $\bar{U}_N$ is given in (A1). Let us now look at θ-marginal of this density, which can be written as

$$\begin{aligned} \pi _\Theta (\theta ) \propto \int e^{-\sum _{i=1}^N U(\theta , \sqrt{N} z_i) - \frac{1}{2} \Vert \bar{v}^z\Vert ^2} {\textrm{d}}z_x {\textrm{d}}\bar{v}. \end{aligned}$$

where $z_x = (z_1, \ldots , z_N) \in {\mathbb {R}}^{N d_x}$. Note now that, integrating out $\bar{v}$, using a change of variables $x_i' = \sqrt{N} z_i$, and setting $x' = (x_1', \ldots , x_N')$, we have

$$\begin{aligned} \pi _\Theta (\theta )&\propto \int _{\mathbb {R}^{Nd_x}} e^{-\sum _{i=1}^N U(\theta , x'_i)} {\textrm{d}}x', \\&\propto \left( \int _{{\mathbb {R}}^{d_x}} e^{-U(\theta , x') } {\textrm{d}}x'\right) ^N\\&= \exp (N \log p_\theta (y)), \end{aligned}$$

since $p_\theta (y) = \int e^{-U(\theta , x)} {\textrm{d}}x$ by definition, where $U(\theta , x) = -\log p_\theta (x, y)$.

### Proof of Proposition 2

Note that, by **H**1, U(θ,x) is jointly μ-strongly convex. Let $\kappa (\theta ) = - \log p_\theta (y)$. Using the Prékopa-Leindler inequality for strongly log-concave distributions ((Saumard and Wellner 2014), Theorem 3.8), we can see that

$$\begin{aligned} \langle \theta - \theta ', \nabla \kappa (\theta ) - \nabla \kappa (\theta ') \rangle \ge \mu \Vert \theta - \theta '\Vert ^2. \end{aligned}$$

The bound now follows using Lemma A.8 from Altschuler and Chewi (2024).

### Proof of Proposition 3

By Proposition A.1, the rescaled process $(\widetilde{{{\textbf{Z}}}}_t,\widetilde{{{\textbf{V}}}}_t^z)$ solves the standard underdamped SDE

C5 $$\begin{aligned} \begin{aligned} {\textrm{d}}\widetilde{{{\textbf{Z}}}}_t&= \widetilde{{{\textbf{V}}}}_t^{z}{\textrm{d}}t,\\ {\textrm{d}}\widetilde{{{\textbf{V}}}}_t^z&= -\widetilde{\gamma }\widetilde{{{\textbf{V}}}}_t^z{\textrm{d}}t - N \nabla \bar{U}_N(\widetilde{{{\textbf{Z}}}}_t){\textrm{d}}t + \sqrt{2\widetilde{\gamma }}\,{\textrm{d}}\widetilde{{{\textbf{B}}}}_t, \end{aligned} \end{aligned}$$

with $\widetilde{\gamma }=\sqrt{N}\gamma$. By Lemmas A.2 and A.3, $\bar{U}_N$ is μ-strongly convex and L-gradient-Lipschitz, hence $N\bar{U}_N$ is Nμ-strongly convex and NL-gradient-Lipschitz. Define

$$ \widetilde{\mu }:=N\mu ,\qquad \widetilde{L}:=NL. $$

The condition $\gamma \ge \sqrt{\mu +L}$ implies

$$ \widetilde{\gamma }=\sqrt{N}\gamma \ge \sqrt{N(\mu +L)}=\sqrt{\widetilde{\mu }+\widetilde{L}}. $$

Therefore Theorem 1 in Dalalyan and Riou-Durand (2020) applies to (C5) and yields, for s≥0,

$$\begin{aligned} W_2\!\left( \mathcal {L}(\widetilde{{{\textbf{Z}}}}_s),{\widetilde{\pi }}_Z\right)&\le \; \sqrt{2}\exp \!\left( -\frac{\widetilde{\mu }}{\widetilde{\gamma }}s\right) \\&\quad \times W_2\!\left( \mathcal {L}(\widetilde{{{\textbf{Z}}}}_0),{\widetilde{\pi }}_Z\right) . \end{aligned}$$

where ${\widetilde{\pi }}_Z$ is the $\widetilde{{{\textbf{Z}}}}$-marginal of $\widetilde{\pi }$ (Lemma A.1).

Now relate rescaled and original time: by definition,

$$ \widetilde{{{\textbf{Z}}}}_{t/\sqrt{N}} =\big ({\boldsymbol{\theta }}_{t},N^{-1/2}{{\textbf{X}}}_t^1,\ldots ,N^{-1/2}{{\textbf{X}}}_t^N\big )^\intercal . $$

Hence the θ-component of $\widetilde{{{\textbf{Z}}}}_{t/\sqrt{N}}$ is ${\boldsymbol{\theta }}_t$, and the θ-marginal of ${\widetilde{\pi }}_Z$ is $\pi _\Theta$. We note

$$\begin{aligned} W_2\!\left( \mathcal {L}({\boldsymbol{\theta }}_t),\pi _\Theta \right) \le W_2\!\left( \mathcal {L}(\widetilde{{{\textbf{Z}}}}_{t/\sqrt{N}}),{\widetilde{\pi }}_Z\right) . \end{aligned}$$

Using $$s=t/\sqrt{N}$$ gives

$$ \frac{\widetilde{\mu }}{\widetilde{\gamma }}\,\frac{t}{\sqrt{N}} =\frac{N\mu }{\sqrt{N}\gamma }\,\frac{t}{\sqrt{N}} =\frac{\mu }{\gamma }t. $$

Therefore

$$\begin{aligned} W_2\!\left( \mathcal {L}({\boldsymbol{\theta }}_t),\pi _\Theta \right) \le \sqrt{2}\exp \!\left( -\frac{\mu }{\gamma }t\right) W_2\!\left( \mathcal {L}(\widetilde{{{\textbf{Z}}}}_0),\widetilde{\pi }_Z\right) . \end{aligned}$$

Finally, with $$\bar{Z}_\star \sim {\widetilde{\pi }}_Z$$,

$$ W_2\!\left( \mathcal {L}(\widetilde{{{\textbf{Z}}}}_0),{\widetilde{\pi }}_Z\right) \le \mathbb {E}\!\left[ \Vert \widetilde{{{\textbf{Z}}}}_0-\bar{Z}_\star \Vert ^2\right] ^{1/2}, $$

which is exactly the stated bound (up to the same notation $\widetilde{{{\textbf{Z}}}}_0\equiv {{\textbf{Z}}}_0$ used in the proposition).

### Proof of Theorem 1

By Proposition A.1, we can rewrite KIPLD as the standard underdamped diffusion with stationary measure

$$\begin{aligned} \widetilde{\pi }(z, v) \propto e^{-N \bar{U}_N(z) - \Vert v\Vert ^2/2}. \end{aligned}$$

We will now look at the standard kinetic Langevin Monte Carlo discretisation of the SDE and show that this scheme coincides with KIPLMC1 to utilize the bounds in Dalalyan and Riou-Durand (2020) directly.

Let us write the Exponential Integrator discretisation of this scheme as we do in B.1,

C6 $$\begin{aligned} \begin{aligned} \widetilde{Z}_{n+1}&= \widetilde{Z}_{n} + \widetilde{\psi }_1^{\tilde{\eta }} \widetilde{V}_n^z - \widetilde{\psi }_2^{\tilde{\eta }} N \nabla \bar{U}_N(\widetilde{Z}_n)+ \sqrt{2 \tilde{\gamma }} \zeta _{n+1}',\\ \widetilde{V}_{n+1}^z&= \widetilde{\psi }_0^{\tilde{\eta }} \widetilde{V}_n^z - \widetilde{\psi }_1^{\tilde{\eta }} N \nabla \bar{U}_N(\widetilde{Z}_n) + \sqrt{2 \tilde{\gamma }} \zeta _{n+1}. \end{aligned} \end{aligned}$$

Since the function $z \mapsto N \bar{U}_N(z)$ is Nμ strongly convex and *NL*-gradient-Lipschitz, for $\tilde{\gamma } \ge \sqrt{N\mu + N L}$ and $\tilde{\eta } \le \mu / (4 \tilde{\gamma } L)$, Dalalyan and Riou-Durand (2020, Theorem 2) directly implies that

$$\begin{aligned} W_2(\nu _n, {\widetilde{\pi }}_Z)&\le \sqrt{2} \mathbb {E}[\Vert Z_0-\bar{Z}_\star \Vert ^2]^{1/2} \left( 1 - \frac{0.75 \tilde{\mu } \tilde{\eta }}{\tilde{\gamma }}\right) ^n \\&\quad + \frac{\tilde{\eta }\tilde{L}}{\tilde{\mu }}\sqrt{2d_z}, \end{aligned}$$

where $\nu _n:=\mathcal {L}({\widetilde{Z}}_n)$ denotes the law of the position iterate in (C6), ${\widetilde{\pi }}_Z$ denotes the position marginal of the stationary measure in Lemma A.1, and $\bar{Z}_\star \sim {\widetilde{\pi }}_Z$. We now recall from B.1, that $\tilde{\eta }=\eta /\sqrt{N}$ and $\tilde{\gamma }=\sqrt{N}\gamma$, from which we obtain,

$$\begin{aligned} W_2(\nu _n, {\widetilde{\pi }}_Z) \le C_1\left( 1-\frac{0.75 \mu \eta }{\gamma }\right) ^n + \eta C_2, \end{aligned}$$

where,

$$\begin{aligned} C_1&= \sqrt{2} {\mathbb {E}}[\Vert Z_0-\bar{Z}_\star \Vert ^2]^{1/2}, \qquad \bar{Z}_\star \sim {\widetilde{\pi }}_Z\\ C_2&=\sqrt{2}\frac{L}{\mu } \sqrt{\frac{d_z}{N}}. \end{aligned}$$

The rescaling leaves the θ-coordinate unchanged, so the θ-component of ${\widetilde{Z}}_n$ is $\theta _n$. Consequently, $\mathcal {L}(\theta _n)$ and $\pi _\Theta$ are the θ-marginals of $\nu _n$ and ${\widetilde{\pi }}_Z$, respectively. Since the coordinate projection $z\mapsto \theta$ is 1-Lipschitz, $W_2(\mathcal {L}(\theta _n),\pi _\Theta )\le W_2(\nu _n,{\widetilde{\pi }}_Z)$. Applying this inequality and the triangle inequality, we obtain

$$\begin{aligned} \mathbb {E}[\Vert \theta _n-\bar{\theta }_\star \Vert ^2]^{1/2}&= W_2(\mathcal {L}(\theta _n), \delta _{\bar{\theta }_\star })\\&\le W_2(\mathcal {L}(\theta _n), \pi _\Theta ) + W_2(\pi _\Theta , \delta _{\bar{\theta }_\star })\\&\le C_1 \left( 1-\frac{0.75 \mu \eta }{\gamma }\right) ^n + \eta C_2 + \sqrt{\frac{C_3}{N}}, \end{aligned}$$

where the last term is bounded by Proposition 2 with $C_3=d_\theta /\mu$.

### Proof of Theorem 2

Again, we consider our rescaled standard underdamped diffusion, given by (A2) and seek to directly apply the results from Monmarché (2021) for our scheme.

We now write the splitting scheme as in B.2,

C7 $$\begin{aligned} \widetilde{Z}_{n+1}= &  \widetilde{Z}_n + \tilde{\eta } (\tilde{\delta } \widetilde{V}^z_n + \sqrt{1-\tilde{\delta }^2} \xi _{n+1})- \frac{\tilde{\eta }^2}{2}N \nabla \bar{U}_N(\widetilde{Z}_n)\nonumber \\ \widetilde{V}^z_{n+1}= &  \tilde{\delta }^2 \widetilde{V}_n^z - \frac{\tilde{\eta }\tilde{\delta }}{2}(N\nabla \bar{U}_N(\widetilde{Z}_n) + N \nabla \bar{U}_N (\widetilde{Z}_{n+1}) ) \nonumber \\ &  \quad + \sqrt{1-\tilde{\delta }^2}(\tilde{\delta }\xi _{n+1} + \xi _{n+1}^\prime ), \end{aligned}$$

where $$\tilde{\delta }=e^{-\tilde{\gamma }\tilde{\eta }/2}$$. Set

$$\begin{aligned} {\widetilde{\mu }}&:=N\mu ,&{\widetilde{L}}&:=NL,\\ {\widetilde{\kappa }}&:=\frac{{\widetilde{\mu }}}{3{\widetilde{\gamma }}},&A_N&:=\sqrt{3}\bigl (\sqrt{NL}\vee (NL)^{-1/2}\bigr ). \end{aligned}$$

By Lemmas A.2 and A.3, the potential $N\bar{U}_N$ is ${\widetilde{\mu }}$-strongly convex and has ${\widetilde{L}}$-Lipschitz gradient. Moreover,

$$\begin{aligned} {\widetilde{\gamma }}\ge 2\sqrt{{\widetilde{L}}}&\quad \Longleftrightarrow \quad \gamma \ge 2\sqrt{L},\\ {\widetilde{\eta }}\le \frac{{\widetilde{\mu }}}{33{\widetilde{\gamma }}^3}&\quad \Longleftrightarrow \quad \eta \le \frac{\mu }{33\gamma ^3},\\ 1-{\widetilde{\eta }}{\widetilde{\kappa }}&=1-\frac{\eta \mu }{3\gamma }=:q. \end{aligned}$$

Indeed, $\mu \le L$ and the stated parameter restrictions give $0<\eta \mu /(3\gamma )\le \mu ^2/(99\gamma ^4)\le 1/1584$, so q∈(0,1). Let ${\widetilde{P}}$ be the transition kernel of (C7), let ${\widetilde{\pi }}_{{\widetilde{\eta }}}$ be its invariant probability measure on phase space, let ${\widetilde{\pi }}$ be the invariant probability measure of the diffusion in Lemma A.1, and let ${\widetilde{\pi }}_Z$ be the position marginal of ${\widetilde{\pi }}$. Theorem 1 of Monmarché (2021) gives, for all $\rho ,\sigma \in \mathcal {P}_2(\mathbb {R}^{2d_z})$,

C8 $$\begin{aligned} W_2(\rho {\widetilde{P}}^n,\sigma {\widetilde{P}}^n) \le A_Nq^{n/2}W_2(\rho ,\sigma ). \end{aligned}$$

Proposition 11 of the same reference, applied with p=2, gives

C9 $$\begin{aligned} b_N&:=W_2({\widetilde{\pi }}_{{\widetilde{\eta }}},{\widetilde{\pi }})\nonumber \\&\le {\widetilde{\eta }}\sqrt{d_z}\, \frac{2A_N{\widetilde{K}}}{{\widetilde{\kappa }}} =\eta \sqrt{d_z}\,\frac{6\gamma {\widetilde{K}}}{N\mu }\,A_N =:B_N, \end{aligned}$$

where

$$\begin{aligned} {\widetilde{K}} :={\widetilde{L}}\left( 1+e^{{\widetilde{L}}{\widetilde{\eta }}^2} \left( \frac{{\widetilde{\eta }}}{6} +\frac{{\widetilde{\eta }}^2{\widetilde{L}}}{24}\right) \right) \left( 1+\frac{{\widetilde{\eta }}{\widetilde{L}}}{2\sqrt{{\widetilde{\mu }}}}\right) . \end{aligned}$$

Substituting ${\widetilde{L}}=NL$, ${\widetilde{\eta }}=\eta /\sqrt{N}$, and ${\widetilde{\mu }}=N\mu$ into ${\widetilde{K}}$ yields the exact identity

$$\begin{aligned} {\widetilde{K}}&=NL\left( 1+e^{L\eta ^2} \left( \frac{\eta }{6\sqrt{N}}+\frac{\eta ^2L}{24}\right) \right) \\&\quad \times \left( 1+\frac{\eta L}{2\sqrt{\mu }}\right) . \end{aligned}$$

Define

$$\begin{aligned} K:=L\left( 1+e^{L\eta ^2} \left( \frac{\eta }{6}+\frac{\eta ^2L}{24}\right) \right) \left( 1+\frac{\eta L}{2\sqrt{\mu }}\right) . \end{aligned}$$

Since N≥1,

$$\begin{aligned} {\widetilde{K}}&\le NK,\\ A_N&\le \sqrt{3N}\bigl (\sqrt{L}\vee L^{-1/2}\bigr ). \end{aligned}$$

For conciseness, set $M_L:=\sqrt{L}\vee L^{-1/2}$ and define

$$\begin{aligned} G_\eta&:=\frac{6\gamma K}{\mu }M_L\\&=\frac{6\gamma L}{\mu }M_L \left( 1+\frac{\eta L}{2\sqrt{\mu }}\right) \\&\quad \times \left( 1+e^{L\eta ^2} \left( \frac{\eta }{6}+\frac{\eta ^2L}{24}\right) \right) . \end{aligned}$$

To make the implicit constant in Theorem 2 uniform in η, set

$$\begin{aligned} \eta _0&:=\frac{\mu }{33\gamma ^3},\\ G_\star&:=\frac{6\gamma L}{\mu }M_L\left( 1+\frac{\eta _0L}{2\sqrt{\mu }}\right) \\&\quad \times \left( 1+e^{L\eta _0^2} \left( \frac{\eta _0}{6}+\frac{\eta _0^2L}{24}\right) \right) . \end{aligned}$$

Every factor in $G_\eta$ is nondecreasing for $\eta \ge 0$. Hence the restriction $0<\eta \le \eta _0$ gives $G_\eta \le G_\star$, where $G_\star$ depends only on μ, *L*, and γ. Consequently, (C9) gives

C10 $$\begin{aligned} B_N&\le \eta \sqrt{d_z}\,\frac{6\gamma K}{\mu }\, \sqrt{3N}\,M_L \le \sqrt{3}\,G_\star \eta \sqrt{Nd_z}. \end{aligned}$$

Let $\varphi :=\mathcal {N}(0,\mathbb {I}_{d_z})$ and let $\rho _n$ be the law of the full rescaled phase-space iterate $(\widetilde{Z}_n,{\widetilde{V}}_n^z)$. The initialisation in Theorem 2 and the rescaling in Proposition B.2 imply that

$$\begin{aligned} \rho _0=\nu \otimes \varphi . \end{aligned}$$

Lemma A.1 gives ${\widetilde{\pi }}={\widetilde{\pi }}_Z\otimes \varphi$. Coupling the two identical velocity marginals, and using the position projection for the reverse inequality, gives

$$\begin{aligned} W_2(\rho _0,{\widetilde{\pi }}) =W_2(\nu ,{\widetilde{\pi }}_Z)=:D_0. \end{aligned}$$

Since ${\widetilde{\pi }}_{{\widetilde{\eta }}}$ is invariant for $\widetilde{P}$, (C8), (C9), and two applications of the triangle inequality yield

$$\begin{aligned} W_2(\rho _n,{\widetilde{\pi }})&\le W_2(\rho _0{\widetilde{P}}^n, {\widetilde{\pi }}_{{\widetilde{\eta }}}{\widetilde{P}}^n)+b_N\\&\le A_Nq^{n/2}W_2(\rho _0,{\widetilde{\pi }}_{{\widetilde{\eta }}})+b_N\\&\le A_Nq^{n/2}(D_0+b_N)+b_N\\&\le A_Nq^{n/2}(D_0+B_N)+B_N\\&\le \sqrt{3}\,M_L\sqrt{N}\,D_0q^{n/2}+B_N\\&\quad +\sqrt{3}\,M_L\sqrt{N}\,q^{n/2}B_N\\&\le \sqrt{3}\,M_L\sqrt{N}\,D_0q^{n/2} +\sqrt{3}\,G_\star \eta \sqrt{Nd_z}\\&\quad +3M_LG_\star \eta N\sqrt{d_z}\,q^{n/2}. \end{aligned}$$

Thus, with

$$\begin{aligned} C_\star :=\max \{\sqrt{3}M_L,\sqrt{3}G_\star ,3M_LG_\star \}, \end{aligned}$$

we have the fully explicit intermediate bound

C11 $$\begin{aligned} W_2(\rho _n,{\widetilde{\pi }})&\le C_\star \sqrt{N}\,D_0q^{n/2}\nonumber \\&\quad +C_\star \eta N\sqrt{d_z}\,q^{n/2}\nonumber \\&\quad +C_\star \eta \sqrt{Nd_z}. \end{aligned}$$

The map $(z,v)\mapsto \theta$ is 1-Lipschitz, the rescaling leaves the θ-coordinate unchanged, and its pushforwards of $\rho _n$ and ${\widetilde{\pi }}$ are $\mathcal {L}(\theta _n)$ and $\pi _\Theta$, respectively. Therefore,

$$\begin{aligned} W_2(\mathcal {L}(\theta _n),\pi _\Theta ) \le W_2(\rho _n,{\widetilde{\pi }}). \end{aligned}$$

Finally, the triangle inequality and Proposition 2 give

$$\begin{aligned} {\mathbb {E}}[\Vert \theta _n-\bar{\theta }_\star \Vert ^2]^{1/2}&=W_2(\mathcal {L}(\theta _n),\delta _{\bar{\theta }_\star })\\&\le W_2(\rho _n,{\widetilde{\pi }}) +\frac{1}{\sqrt{\mu }}\sqrt{\frac{d_\theta }{N}}. \end{aligned}$$

Moreover, since $$q=1-\eta \mu /(3\gamma )\in (0,1)$$,

$$\begin{aligned} q^{n/2}&\le \exp \left( -\frac{\mu \eta n}{6\gamma }\right) . \end{aligned}$$

Combining this inequality with (C11), substituting $d_z=d_\theta +Nd_x$, and absorbing $C_\star$ and $\mu ^{-1/2}$ into an implicit constant gives, with $\mathcal {E}_n:={\mathbb {E}}[\Vert \theta _n-\bar{\theta }_\star \Vert ^2]^{1/2}$,

$$\begin{aligned} \mathcal {E}_n&\lesssim \sqrt{N}\,W_2(\nu ,{\widetilde{\pi }}_Z) \exp \left( -\frac{\mu \eta n}{6\gamma }\right) \\&\quad +\eta N\sqrt{d_\theta +Nd_x} \exp \left( -\frac{\mu \eta n}{6\gamma }\right) \\&\quad +\eta \sqrt{N(d_\theta +Nd_x)} +\sqrt{\frac{d_\theta }{N}}. \end{aligned}$$

The implicit constant depends only on μ, *L*, and γ, and this is the claimed bound.

## Results

### Multi-well experiment

We include the following multi-well experiment to highlight the advantage of the noised θ dynamics, the core difference between the proposed methods and MPGD (Lim et al. 2024). To do this, we consider a synthetic example with a non-convex negative log-marginal-likelihood objective. Consider the joint probability density over $\mathbb {R}$,

$$\begin{aligned} p_\theta (x,y)&= \mathcal {N}(y; x, \sigma ^2_y)p_\theta (x),\\ p_\theta (x)&= \frac{1}{2}\mathcal {N}(x; -\theta ,\sigma ^2_x) + \frac{1}{2}\mathcal {N}(x;\theta ,\sigma _x^2). \end{aligned}$$

For $|y|>\sqrt{\sigma _x^2+\sigma _y^2}$, the marginal likelihood is bimodal as a function of θ. Indeed, integrating out *x* gives

$$\begin{aligned} p_\theta (y) = \frac{1}{2} \mathcal {N}(y;\theta , \sigma _x^2+\sigma _y^2) + \frac{1}{2}\mathcal {N}(y;-\theta , \sigma _x^2 +\sigma _y^2). \end{aligned}$$

In this case, we seek to show that whilst the MPGD algorithm gets stuck in one of the local minima, the KIPLMC1 algorithm escapes. For the experiment, we generate a single observation from the model with θ=1.5, and we fix $\sigma _x^2=1$ and $\sigma _y^2=0.25$. Since $p_\theta =p_{-\theta }$, the values $\theta =\pm 1.5$ are observationally equivalent data-generating parameter values; they need not coincide exactly with the maximisers of the marginal likelihood for the realised observation. In Fig. 5, we show the trace plots of θ together with the associated empirical density plots, compared with these two reference values and the target θ-density proportional to $p_\theta (y)^N$, respectively. For sake of comparison, we also compare the dynamics of KIPLMC1 and KIPLMC2 on the marginal dynamics, i.e. we consider KIPLD to be given simply as

$$\begin{aligned} \textrm{d}{\boldsymbol{\theta }}_t&= {{\textbf{V}}}_t^\theta \textrm{d}t,\\ \textrm{d}{{\textbf{V}}}^\theta _t&= -\gamma {{\textbf{V}}}_t^\theta \,\textrm{d}t -\nabla _\theta \kappa ({\boldsymbol{\theta }}_t)\,\textrm{d}t + \sqrt{\frac{2\gamma }{N}}\,\textrm{d}{{\textbf{B}}}_t, \end{aligned}$$

for an $\mathbb {R}^{d_\theta }$-valued Brownian motion ${{\textbf{B}}}_t$. Recalling from Proposition 1 that $\kappa (\theta )=-\log p_\theta (y)$, the invariant θ-density of these marginal dynamics is proportional to $p_\theta (y)^N$. In Fig. 5, all methods approach at least one of the two wells, but only the “noised” algorithms KIPLMC1, KIPLMC2 and IPLA reliably explore both wells.

### Computational cost comparison

We further perform an additional experiment on the Sect. 5.1 example, to compare MSE against computational cost. Indeed, this allows for more thorough comparisons with the methods, as KIPLMC1 generates correlated noise, KIPLMC2 performs a gradient correction and MPGD does both. Further all these methods require twice as much space in memory, when compared to PGD and IPLA, due to the presence of the momentum variables.

In Fig. 6, we observe the faster initial convergence rate of the momentum based methods, despite their greater computational cost. Further, we observe that the increased cost of noising the θ-dynamics in our proposed methods, is negligible when compared to a second gradient evaluation per iteration, required by MPGD and KIPLMC2.

### Area between the curves calculation (ABC)

To compare the behaviour of the different algorithms, we employ a measure that quantifies how accurately and quickly each algorithm converges to the true solution, namely the ABC, as in Lim et al. (2024). Consider $c^k:\mathbb {N}\rightarrow \mathbb {R}$ and $c^m:\mathbb {N}\rightarrow \mathbb {R}$ to be the accuracy curves of KIPLMC2 and IPLA respectively, i.e. for $\{\theta _n\}_{n=1}^\infty$ generated by the KIPLMC2 algorithm, $c^k(n)=\Vert \theta _n -\bar{\theta }^\star \Vert$. The ABC is given as,

$$\begin{aligned} \sum _{i=1}^M w(i) (c^m(i)-c^k(i)), \end{aligned}$$

where *M* is the total number of time-steps and *w* acts as a weighting function w(i)=2i/(M(M+1)), matching Kuntz et al. (2023). Hence, the ABC computes a signed, weighted area between the error curves of IPLA and KIPLMC2. In particular, when $c^m$ is dominated by $c^k$, the ABC is negative, whilst positive in the converse case.

### Error metrics for the BNN

Recall that the output of our model for latent variable x=(w,v), image features *f* is,

$$\begin{aligned} p(l|f, x) \propto \exp \left( \sum _{j=1}^{40} v_{lj} \tanh \left( \sum _{i=1}^{784} w_{ji} f_i\right) \right) . \end{aligned}$$

To normalise the outputs of the RHS over the labels we apply softmax to the estimates for all *l*. Denote this as *g*(*l*|*f*, *x*) and we give the output of the model as

$$\begin{aligned} \hat{l}(f|x) = {\mathop {\textrm{argmax}}\limits _{l\in \{0,1\}}}\, g(l|f,x). \end{aligned}$$

For the BNN in 5.3 we use two metrics to evaluate the performance of our models, which we evaluate on test set of 200 images and labels $\mathcal {Y}_\text {test}$. In particular we use a relative error metric, measuring the percentage accuracy on the test set,

$$\begin{aligned} \frac{1}{|\mathcal {Y}_\text {test}|}\sum _{(f,l)\in \mathcal {Y}_\text {test}} |l - \hat{l}(f|x)|. \end{aligned}$$

Following Kuntz et al. (2023), our second metric is the log pointwise predictive density (LPPD),

$$\begin{aligned} \frac{1}{|\mathcal {Y}_\text {test}|}\sum _{(f,l)\in \mathcal {Y}_\text {test}} \log g(l|f,x). \end{aligned}$$

It is the average log probability assigned by the model to the correct test label; larger values indicate better predictive performance.
